# Supplementary material for: Magneto-responsive chain-like arrangements of size-tuned and cobalt-doped ferrites derived from silica-encapsulated precursors
Source: Commun Chem. 2025 Oct 13;8:305. doi: 10.1038/s42004-025-01730-9 (PMC12518544; doi:10.1038/s42004-025-01730-9)
Supplement: Supplementary file 2 — Supplementary Information [file 42004_2025_1730_MOESM2_ESM.pdf]

Supplementary Information

Magneto-responsive Chain-like Arrangements of Size-tuned and Cobalt-doped Ferrites derived from Silica-encapsulated Precursors

Maria Weißpflog[1]*, Julia Kabelitz[1], Birgit Hankiewicz[1]*

[1] University of Hamburg, Institute of Physical Chemistry, Grindelallee 117, 20146 Hamburg, Germany

* Corresponding authors: Maria Weißpflog (maria.weisspflog@uni-hamburg.de); Birgit Hankiewicz (birgit.hankiewicz@uni-hamburg.de).

Content

Supplementary Section 1. Akaganeite Precursor

Supplementary Section 2. Surface Modification of Akaganeite Precursors with PAA

Supplementary Section 3. Akaganeite Precursors with Different Silica Shell Thicknesses

Supplementary Section 4. Size-Dependent Synthesis and Arrangement of Cobalt Ferrite Chains: Overview

Supplementary Section 5. Size-Dependent Synthesis and Arrangement of Cobalt Ferrite Chains: Temperature Effect

Supplementary Section 6. Size-Dependent Synthesis and Arrangement of Cobalt Ferrite Chains: Silica Shell Thickness

Supplementary Section 7. Size-Dependent Synthesis and Arrangement of Cobalt Ferrite Chains: Mechanism and Summarized Effects

Supplementary Section 8. Hysteresis of Self-Arranged Nanochains

Supplementary Section 9. SAXS Measurements

Supplementary Section 10. Additional Experimental Data

Supplementary Section 11. References

Supplementary Section 1. Akaganeite Precursor

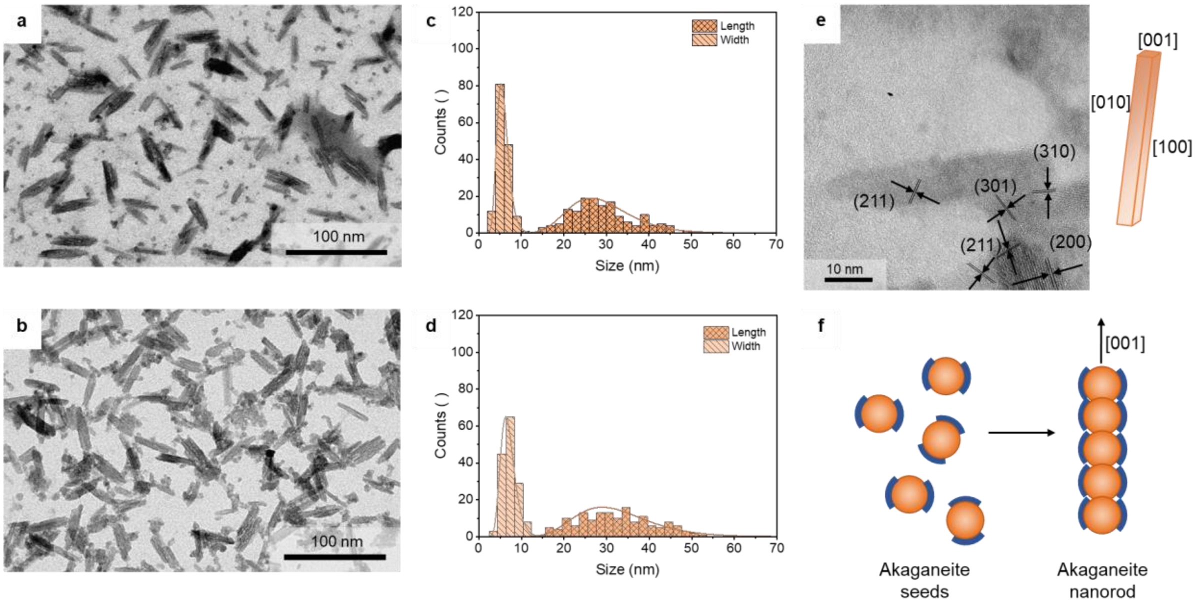

Supplementary Figure 1.1. Morphology of the nanorod precursor. TEM images of two different batches of particle suspensions **a)** aka1 and **b)** aka2, and **c-d**) the corresponding histograms with log-normal distribution curves with an average nanorod length of 32 nm and a width of 7 nm, depict the successful synthesis of nanorod precursor suspensions. Nanorods with the following average values for length x width are shown in Supplementary Figures 1.1a and 1.1b: aka1 = (31 ± 8) nm x (7 ± 2) nm and aka2 = (33 ± 9) nm x (7 ± 2) nm. Considering a cylindrical geometry, the nanoparticle samples aka1 and aka2 have surface areas of approximately 750 nm^2 and 800 nm^2 per particle, respectively. **e**) The schematic illustration of the nanorod clarifies the anisotropic growth in the [001] direction, as discussed in the HR-TEM analysis using inverse Fast Fourier Transformation, where the major axis of the nanorods was perpendicular to the {200} set of crystal planes (explanation later in Supplementary Section 1.3). **f**) The anisotropic growth is promoted not only by the double chain orientation but also by the presence of phosphate ions (illustrated in blue) on the (*hk*0) faces of the initially synthesized akaganeite seeds (orange). In particular, phosphate ions serve to prevent akaganeite from oxidation to hematite by hindering the agglomeration of various nanorods along the [010] and [100] directions.

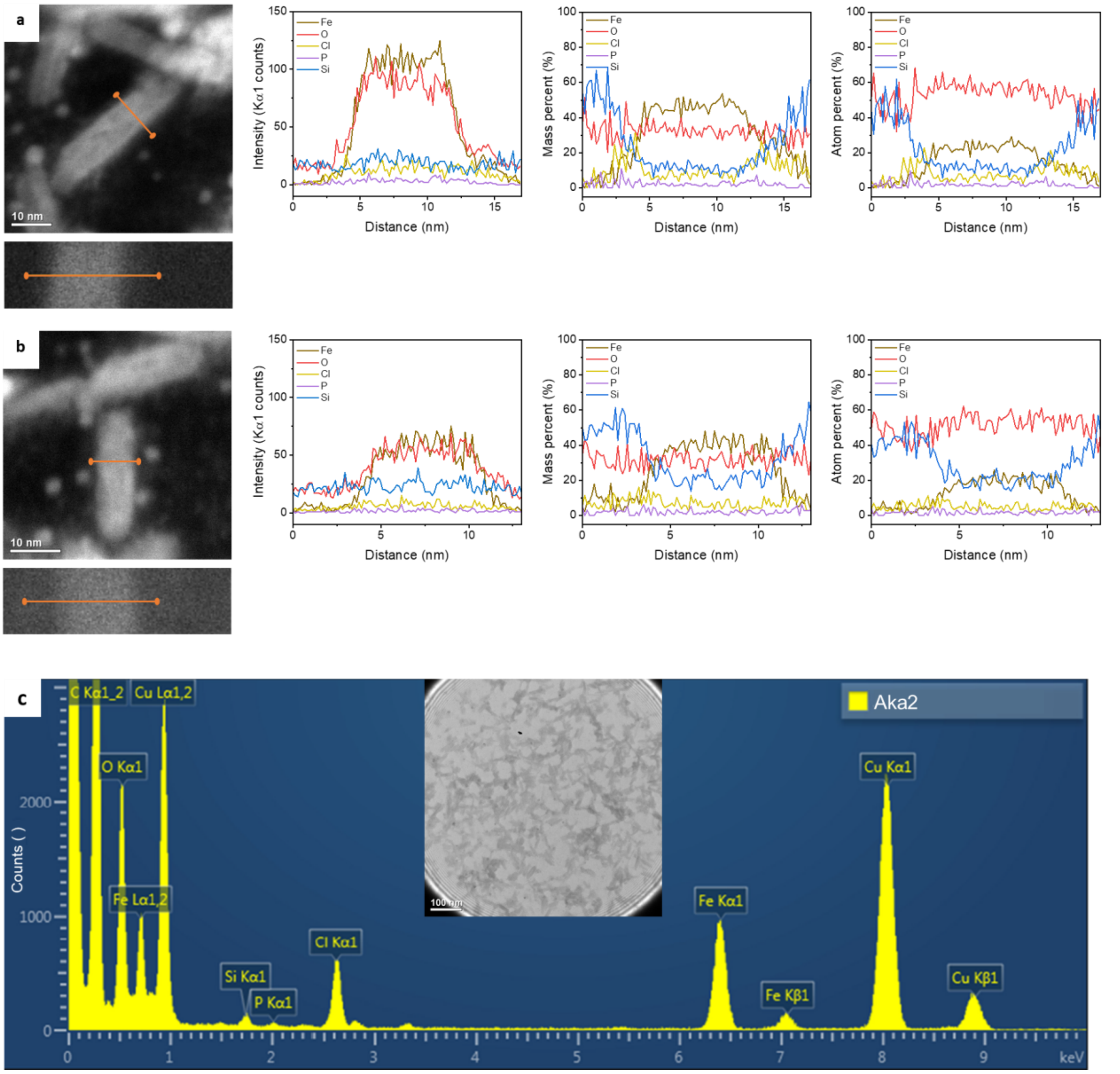

Supplementary Figure 1.2. Line scan profiles and sum spectra of the nanorod precursor sample aka2. TEM images and corresponding EDX line-scanning profiles across an individual akaganeite nanorod, with the Kα intensity, mass percent, and atom percent for the elements Fe, O, Cl, P, and Si displayed from left to right, given a Fe to Cl ratio of around **a**) 4.2:1 and **b**) 4.9:1. **c**) EDX sum spectrum of a typical nanorod section as depicted in the inset, giving a Fe to Cl ratio of around 2.8:1, which is much lower than for the line scans because of the NaCl impurities incorporated in the data (see later Supplementary Figure 1.3). The Si/Fe ratio is 0.09, which is interpreted as background for the interpretation of the Si/Fe ratios of the silica-functionalized particles.

Supplementary Note 1.1. Influence of the additive Na_2HPO_4

The quality of the precursor solution depends on several parameters, including the pH value, the purity of Fe(III)-chloride hexahydrate, and the state of matter of Na_2HPO_4, as demonstrated in previous work.[1,2] Subsequent magnetic particle quality and quantity regarding size distribution and yield using the co-precipitation method following a hydrothermal step are strongly associated with this.[1]

Additionally, Na_2HPO_4 is required to prevent akaganeite from transforming into hematite. Ozaki *et al.* investigated the hematite formation through an akaganeite intermediate step *via* forced hydrolysis of ferric chloride in aqueous solution, depending on the concentration of Na_2HPO_4.[3] It was also demonstrated that the presence of phosphate significantly influences the particle morphology, transitioning from spherical to spindle-like, as the ions bind preferentially to (*hk*0) faces retarding the growth along the *c*-axis.[4,5] The hematite formation can be explained *via* two different mechanisms.[6] Frandsen *et al.* predicted an oriented-aggregation process of the intermediate akaganeite, while Itoh and Sugimoto hypothesized a dissolution-recrystallization process of the akaganeite particles.[4,5] It was also demonstrated that the presence of phosphate significantly influences the particle morphology, transitioning from spherical to spindle-like. At concentrations exceeding $4 \cdot 10^{-4}$ M Na_2HPO_4, mixed phases of hematite and akaganeite are formed. With further increase, the formation of hematite is prevented due to the inhibiting effect on the growth rate or dissolution rate, respectively. Considering both mechanisms, phosphate ions should disrupt these processes by interacting with the surface of the nuclei or sub-units of akaganeite, except for the (001) plane.[7] The presence of phosphate on the surface is demonstrated by the maxima in the $K\alpha$ intensity of Phosphorus in the line scan profiles at distances of 2.5 nm and 12.5 nm, reaching up to 10 atom% (Supplementary Figure 1.2). The subsequent growth mechanism of the nanorods can then preferentially occur on the unblocked facets of the akaganeite sub-units along the *c*-axis in dependence on the phosphate concentration, as proposed by Hijnen *et al.*[8] and Bailey *et al.*[9] The proposed formation mechanism is illustrated in Supplementary Figure 1.1f, as also demonstrated by Purbia *et al.*, but using other surfactants.[10] In addition, akaganeite is recognized for its high phosphate adsorption capacity, which is attributed to its large surface area. However, "The specific sorption mechanisms through which the adsorption of phosphate takes place on these adsorbents are still not clear. This is because adsorption is a complicated process and depends on several interactions, such as electrostatic and hydrophobic interactions.", as summarized by Zhao *et al.*[11] In their review, the adsorption characteristics are investigated in relation to the synthesis methods and particle morphologies.[11] It can also be hypothesized that bound chloride ions could prevent phosphate from binding, thereby disrupting the anisotropic growth mechanism of akaganeite rods and preventing hematite from undergoing a phase transformation. This effect should be minimal within the investigated concentration range, as the chloride ions are strongly bound within the tunnel structures of akaganeite.

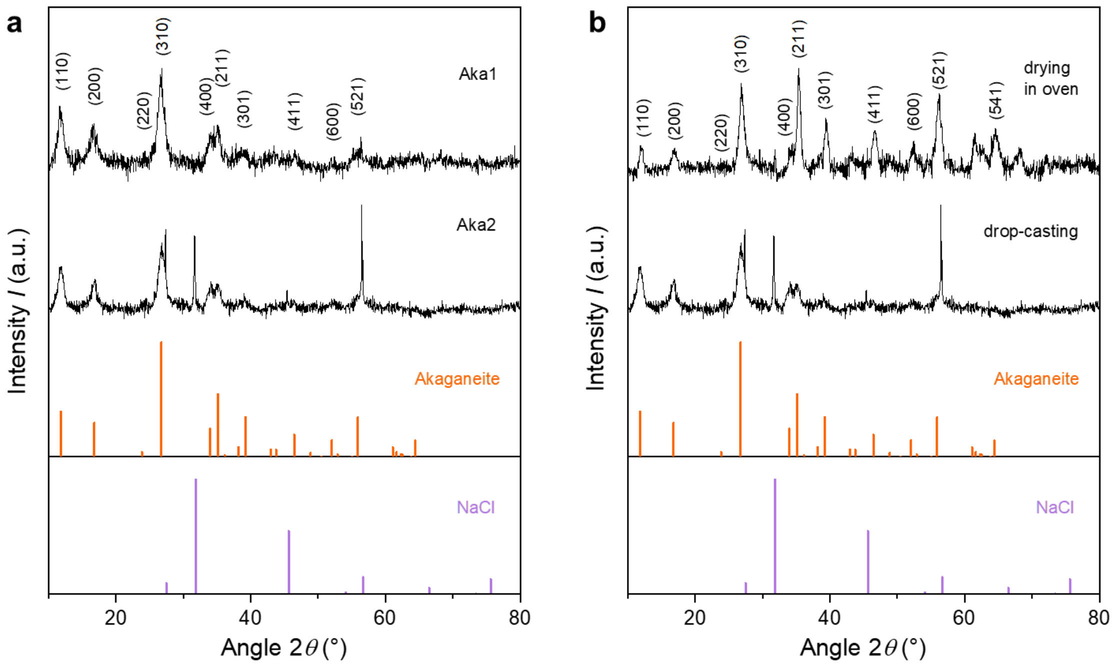

Supplementary Figure 1.3. Composition of the nanorod precursor. a) Diffraction patterns of the two different batches clarified the successful synthesis of akaganeite nanorod precursor suspensions. **b**) The measurement of the powder allows for the determination of intensity ratios of the (310) and (211) reflections in comparison to the drop-casted sample, as no preferred orientation of the nanorods occurs on the silicon wafer.

Supplementary Table 1.1. The (*hkl*) planes, d-spacings d_{hkl}, and corresponding intensities *I* of the reflexes of the samples aka1 and aka2 obtained from SAED and XRD patterns are summarized in comparison to the akaganeite reference.

	**Reference sample (00-034-1266)**			**Aka1 (drop-casting)**		**Aka2 (drop-casting)**			**Aka2 (powder)**	
**Miller indice**	**Angle**	***d*-spacing**	**Intensity**	**Intensity**	**Intensity ratio**	***d*-spacing**	**Intensity**	**Intensity ratio**	**Intensity**	**Intensity ratio**
**(hkl)**	2θ	$d_{(hkl)}$	I_{ref}	I	I/I_{ref}	$d_{(hkl)}$	I	I/I_{ref}	I	I/I_{ref}
	**(°)**		**(%)**	**(%)**			**(%)**		**(%)**	
110	11.8	7.467	40	66.7	1.7	7.50	69.7	1.7	32.1	0.8
200	16.8	5.276	30	51.4	1.7	5.45	52.5	1.7	27.9	0.9
220	23.9	3.728	5	22.1	4.4	3.70	23.9	4.8	14.1	2.8
310	26.7	3.333	100	100.0	1.0	3.41	100	1.0	87.2	0.9
400	34.0	2.634	25	44.0	1.8		49.5	2.0	27.4	1.1
211	35.2	2.550	55	50.0	0.9	2.56	46.2	0.8	100.0	1.8
301	39.2	2.295	35	29.1	0.8	2.31	33.9	1.0	56.6	1.6
411	46.4	1.954	20	25.3	1.3		26.0	1.3	45.4	2.3
600	52.0	1.756	15	21.9	1.5		26.3	1.8	27.3	1.8
521	55.9	1.643	35	29.2	0.8	1.57	32.5	0.9	77.6	2.2
541	64.4	1.446	15	23.5	1.6		19.3	1.3	47.0	3.1

Supplementary Note 1.2. Structure and Composition of the Akaganeite Precursor

The diffraction pattern in Supplementary Figure 1.3a**,** obtained by drop-casting from akaganeite solutions and allowing them to dry at ambient conditions, clearly matches the 2θ angles with JCPDS PDF No. 00-034-1266. A visible amount of sodium chloride is present in aka2. However, the presence of sodium chloride was neglected and not reduced *via* dialysis, as the functionalization with the sodium salt of polyacrylic acid polymers in the subsequent step was associated with a dilution of the sample, and no differences in the PAA modification were observed using the different akaganeite batches.

Through the analysis of the reflection intensities, insights into the crystal orientation with respect to the preferred exposed facets can be obtained.[12] Lattice planes perpendicular to this orientation are expected to exhibit increased intensity. Due to the NaCl impurities in sample aka2, the intensities of the reflexes were normalized to the intensity of the (310) reflex. The intensities of the (220) reflex of both samples varied by a factor of 4.4 to 4.8 in comparison to the reference (Supplementary Table 1.1).

This means that the intensity of the (220) reflex increases by a factor of 4 to 5 compared to the (310) reflex for each akaganeite sample, which differs sufficiently from the reference sample with a factor of 20. Additionally, Almeida *et al.* observed that the intensity ratio of the (310) to (211) reflections in spherical akaganeite particles is comparable.[13] In contrast, in nanorods, the (211) reflection is distinctly more intense, indicating a preferential growth along the [001] direction.[13] A comparable trend in the intensity ratio is also observed in our samples, suggesting a similar predominant growth along the [001] direction. The major axis of the nanorods was perpendicular to the {200} set of crystal planes (Supplementary Figure 1.1e). This is also demonstrated for various one-dimensional nanostructures, such as nanorods, nanowires, and nanotubes, where anisotropic growth is controlled by surfactants, including carbonate, phosphate, and sulfate ions.[7] Conversely, Purbia *et al.* compared the relative intensities, noting that their spherical particles exhibit diffraction patterns with a {310} to {211} intensity ratio similar to that of our akaganeite rods.[10] However, their analysis based on HR-TEM indicated that the (211) planes are preferentially exposed surfaces using tea extracts as a surfactant, which may relate to the effect of the preferred orientation of the rods on the substrate resulting from drop-casting onto a silicon wafer. This could introduce intensity artifacts or missing peaks in the diffraction pattern.[8] Consequently, reflections corresponding to planes in the [001] direction - the rods' growth direction as demonstrated in Supplementary Figure 1.1e - may be affected, and therefore, simple intensity comparisons should be approached with caution. To mitigate this effect, we also performed an XRD measurement on a powder sample that was dried in an oven at 90 °C and ground prior to the measurement. At these temperatures, no phase conversion is assumed.[14] This also aims to exclude any compositional and morphological influences, such as the different scattering abilities of atoms and anisotropic broadening.[12] A clearly intensified reflection of the (211) compared to the (310) is observable, which indicates a nanorod structure and is consistent with the findings of Almeida *et al.*[13] It is suggested that the surfactant is preferentially adsorbed onto the corresponding crystal face, thereby promoting anisotropic growth.

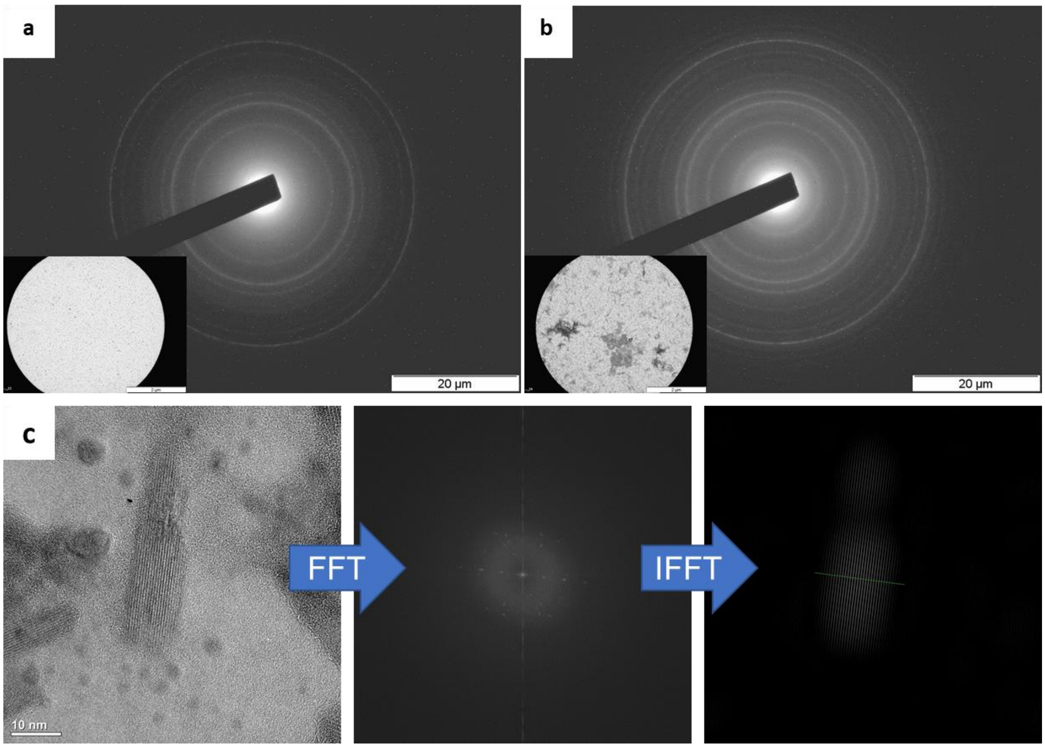

Supplementary Figure 1.4. The analysis of the selected area diffraction patterns of a) aka1, b) aka2, and c) HR-TEM image of aka2 confirms the data obtained from the XRD analysis. The selected area diffraction patterns of **a)** aka1 and **b)** aka2 were examined by measuring the radii of the diffraction rings and determining the *d*-spacings (see Supplementary Table 1.1), which confirms the results of the XRD analysis. **c)** The assignment of the d-spacings in the HR-TEM image of the sample aka2 was performed using a Fast Fourier transformation (FFT). Each spot can be analyzed by an inverse fast Fourier transformation (IFFT) and a corresponding line profile scan. From this, the calculated interplanar spacings can be assigned to the Miller indices, as indicated in Supplementary Figure 1.1e.

Supplementary Section 2. Surface Modification of Akaganeite Precursors with PAA

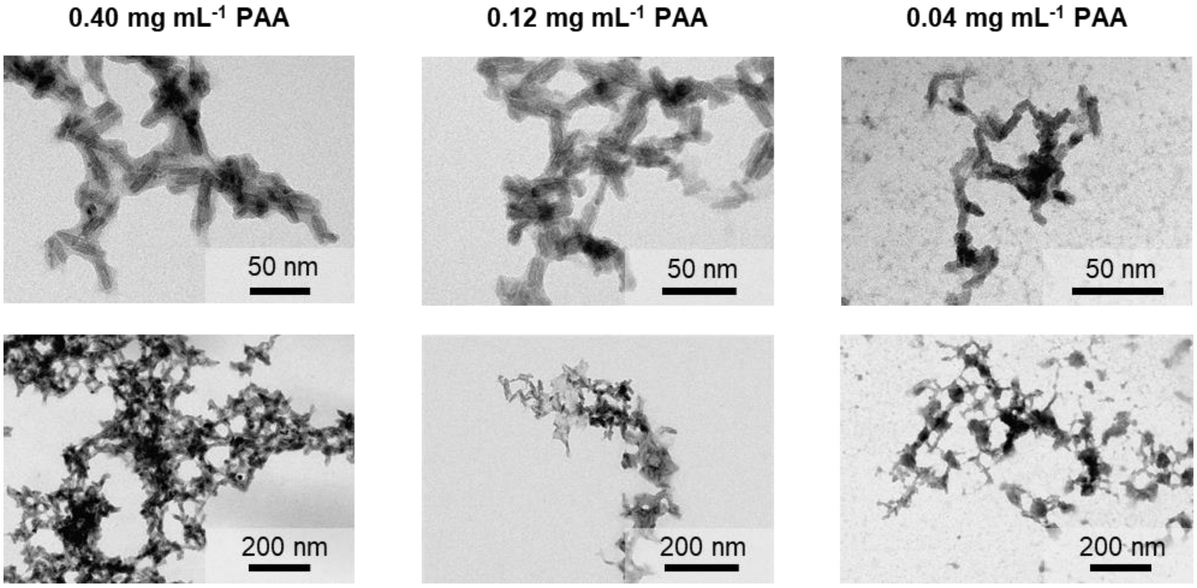

Supplementary Figure 2.1. Morphology of polyacrylic acid polymer-modified nanorod precursors. Different experiments were carried out using high (0.40 mg mL^{-1}), middle (0.12 mg mL^{-1}), or low (0.04 mg mL^{-1}) concentrations of the sodium salt of polyacrylic acid (PAA), meaning a molar amount per surface area of $2.0 \cdot 10^{-28}$ mol nm^{-2}, $1.9 \cdot 10^{-29}$ mol nm^{-2} or $5.2 \cdot 10^{-30}$ mol nm^{-2}. The presence of the PAA polymer is clearly visible in the TEM images at the high polymer concentration. Still, it can also be observed in the middle and low concentrations of the polymer. Furthermore, aggregates formed at high and middle concentrations, leading to particles predominantly assembling into chains, rings, or larger aggregates.

Supplementary Note 2.1. FTIR Spectra of the PAA-modified Precursor

In the FTIR spectra of the akaganeite sample (Supplementary Figure 2.2a), a broad band observed in the range of approximately 3650 – 2750 cm^{-1} corresponds to antisymmetric and symmetric O–H stretching vibrations, while a band around 1620 – 1630 cm^{-1} was ascribed to the H–O–H bending. The spectral features attributed to FeO_6 groups are investigated at wavenumbers ranging from 400 to 1100 cm^{-1}.[15,16] Especially, the bands observed at nearby 867 and 688 cm^{-1} were linked to the –OH bending modes present in β-FeOOH, which are close to those observed by Mei *et al.*.[15] With the functionalization of the akaganeite with PAA, the O–H stretching bands are broadening in the range of approximately 3780 – 2750 cm^{-1} with the PAA concentration. Furthermore, FTIR spectroscopy demonstrated successful surface modification by measuring the characteristic C=O carboxylic acid (1760 – 1690 cm^{-1}) and C–O (1320 – 1210 cm^{-1}) stretching vibrations. The Fe–O bending vibrations are still present after the modification.

Supplementary Note 2.2. XRD Spectra of the PAA-modified Precursor

In the diffraction pattern of a magnified sample modified with a concentration of 0.40 mg mL^{-1} of PAA, it is evident that the akaganeite phase remains unchanged at a high PAA concentration (Supplementary Figure 2.2b). However, traces of NaCl impurities are detected. These impurities may originate from the higher concentration of the ionic sodium-PAA polymer and chloride ions present within the tunnels of the akaganeite structure. With a lower concentration of PAA, the akaganeite can still be modified without the formation of additional sodium chloride (Supplementary Figure 2.2c). Therefore, subsequent syntheses were consistently conducted at 0.12 mg mL^{-1} and 0.04 mg mL^{-1} PAA concentrations.

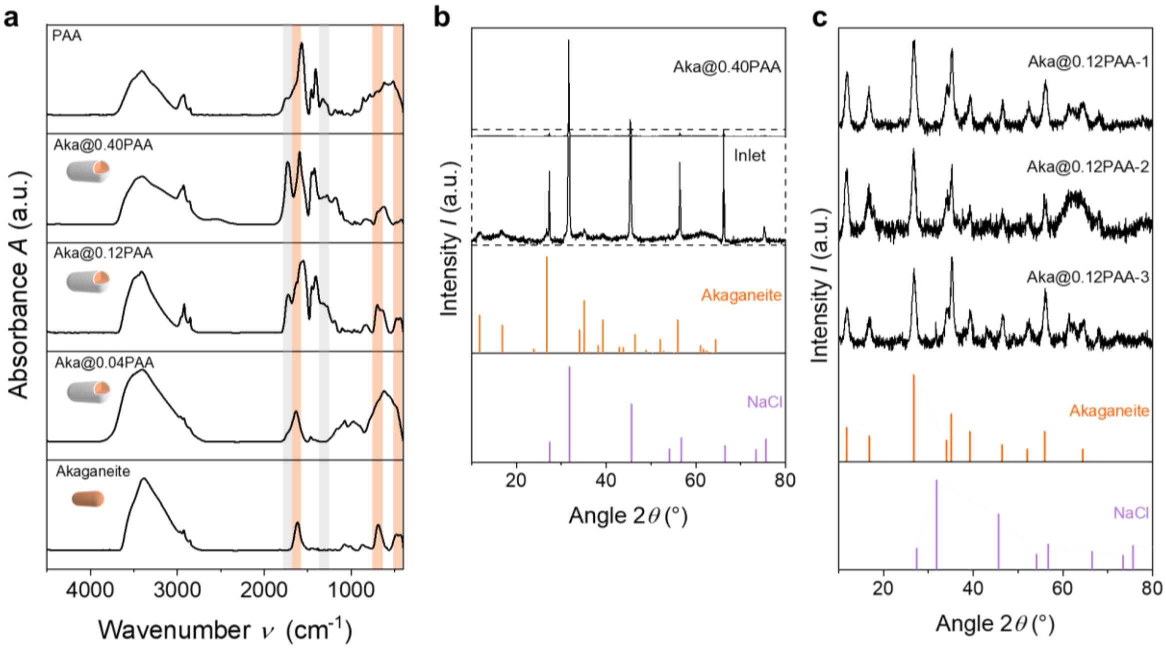

Supplementary Figure 2.2. Successful surface functionalization of the akaganeite nanorods with polyacrylic acid polymer without compositional changes of the akaganeite crystalline phase. a) The O–H- and FeO_6-related stretching and bending vibrations observed via FTIR of the modified dried samples indicate the successful surface functionalization of the akaganeite nanorods with the polyacrylic acid polymer. **b**) The aka@0.40PAA sample exhibits a high level of impurities, including sodium chloride (as shown above). The inlet illustrates the presence of reflexes of akaganeite. **c**) The diffraction patterns of different batches of the akaganeite suspensions modified with 0.12 mg mL^{-1} PAA are comparable. The observed peaks match the akaganeite phase in very good agreement and do not differ from those of the akaganeite suspension (see Supplementary Figure 1.1c). The {211} reflection is distinctly more intense, indicating still a preferential growth along the [001] direction after PAA modification.

Supplementary Section 3. Akaganeite Precursors with Different Silica Shell Thicknesses

Supplementary Note 3.1. Silica Thicknesses analyzed through TEM

For the silica functionalization, akaganeite particles modified with varying concentrations of PAA were employed (Supplementary Figure 3.1a-o). The illustration shown in Supplementary Figure 3.1 depicts the silica thickness d_{SiO2} determined by measuring the width (*w*) of a core-shell particle *via* TEM under the assumption that the PAA shell has no significant effects on the overall thickness of the precursor and that the width of akaganeite corresponds to the average value obtained in Supplementary Section 1. In approximation, the layer thickness was calculated according to Supplementary Equation 3.1:

$$d_{SiO2} \approx \frac{(w_{\text{core-shell-particle}} - w_{\text{akaganeite}})}{2} \qquad 3.1$$

The TEM images of the various silica thicknesses using different PAA modifications are summarized in Supplementary Figure 3.2, and the histograms are given in Supplementary Figure 3.3, where the squares represent the 0.40 mg mL^{-1}, the circles the 0.12 mg mL^{-1}, and the triangles the 0.04 mg mL^{-1} PAA modified suspensions, respectively.

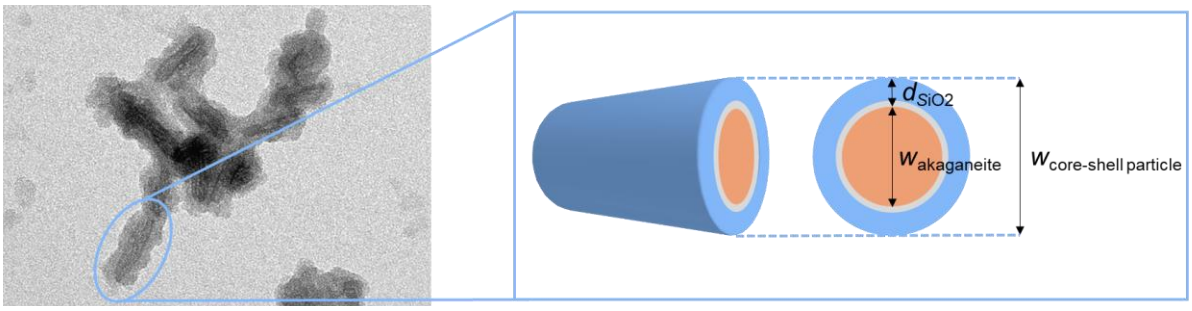

Supplementary Figure 3.1. Calculation of the silica layer thickness. The silica thickness d_{SiO2} was determined by measuring the width (*w*) of a core-shell particle as depicted in the exemplary TEM image and applying Supplementary Equation 3.1.

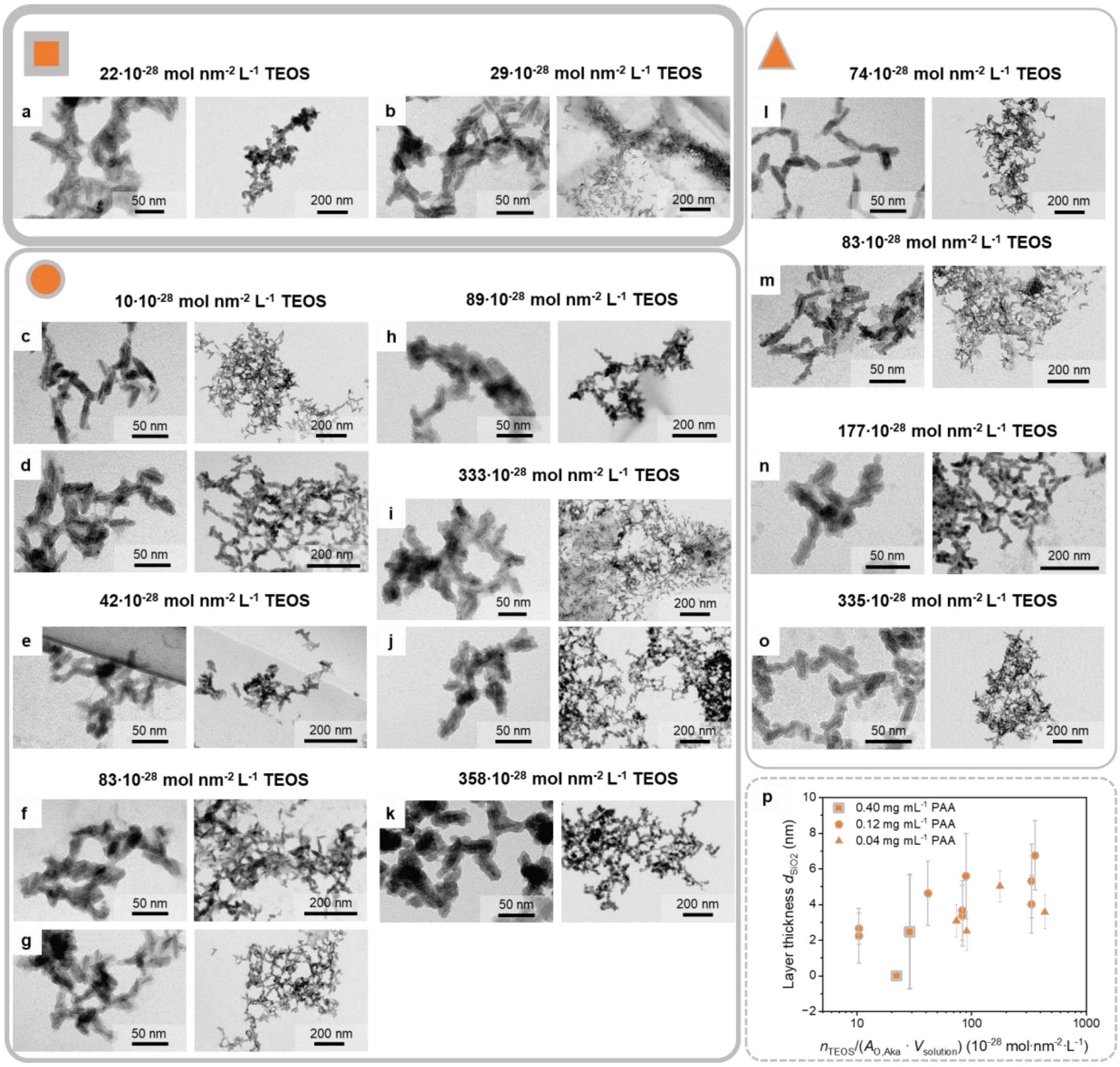

Supplementary Figure 3.2. TEM images of the akaganeite nanorods with different silica thicknesses. The surface modifications of the nanorods with varying amounts of TEOS are depicted for precursors, which are functionalized with **a-b)** 0.40 mg mL^{-1} (squares), **c-k)** 0.12 mg mL^{-1} (circles), or **l-o)** 0.04 mg mL^{-1} (triangles) PAA. (**p**) The thickness of the synthesized layers is shown in dependence on the amount of TEOS per surface area and volume of the reaction solution for all precursors.

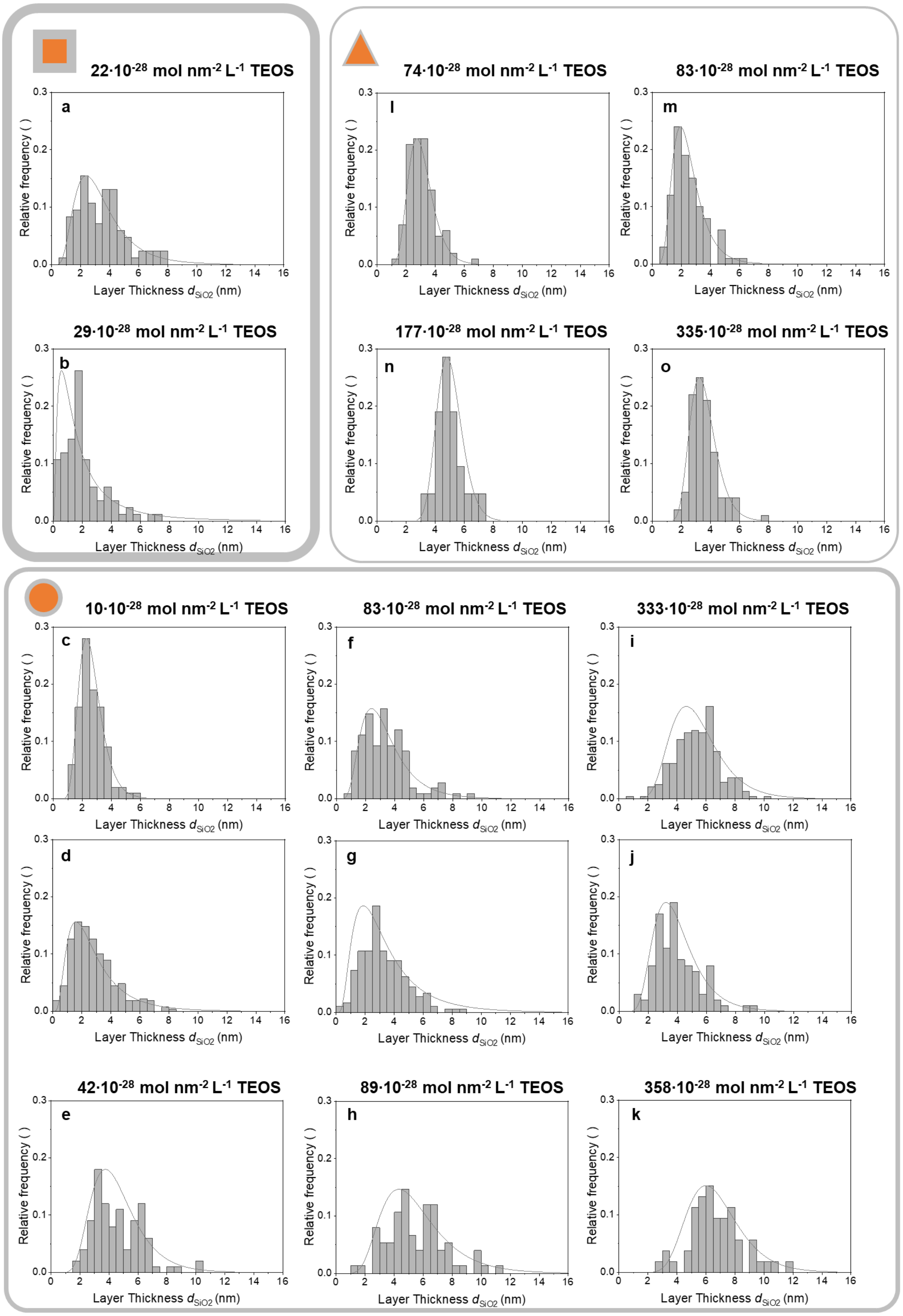

Supplementary Figure 3.3. Histograms of the silica thickness with lognormal distribution curves synthesized with a-b) 0.40 mg mL^{-1} (squares), c-k) 0.12 mg mL^{-1} (circles), or l-o) 0.04 mg mL^{-1} (triangles) PAA-modified akaganeite.

Supplementary Note 3.2. Influence of the PAA Concentration on the Silica Thicknesses

The mass concentration of the nanorods was consistently maintained at 2.6 mg mL^{-1}. The silica-nanorods with 0.40 mg mL^{-1} PAA exhibited pronounced agglomeration, despite the relatively low TEOS concentration per total surface area of the particles, ranging from 20 mmol nm^{-2} L^{-1} to 30 mmol nm^{-2} L^{-1}. A decrease in PAA concentration initially led to a reduced agglomeration after the modified Stöber sol-gel process. Concurrently, an increase in the TEOS concentration per total surface area resulted in a corresponding rise in silica thickness. Silica layers can be formed with thicknesses ranging approximately from 2 nm to 7 nm, depending on the amount of TEOS utilized. Reproducibility tests indicate satisfactory repeatability. Further reduction in the PAA concentration also enabled the synthesis of silica shells, which appear to exhibit less agglomeration. This observation can be attributed to the reduced thickness of the silica shells formed compared to those achieved with akaganeite at a concentration of 0.12 mg mL^{-1}, using the same or a nearby TEOS/surface concentration.

Supplementary Note 3.3. Silica Thickness using HR-TEM, line scan profile, and EDX analysis

The HR-TEM analysis of the silica-functionalized particles (Supplementary Figure 3.4) reveals the presence of akaganeite reflections, which are also observed after the Stöber process. The line scan profiles additionally confirm the presence of Fe signals in the middle of a silica-functionalized particle as well as an increasing intensity of the silicon signal comparing a thin (Supplementary Figure 3.5) and a thick (Supplementary Figure 3.6) silica shell around the akaganeite particle.

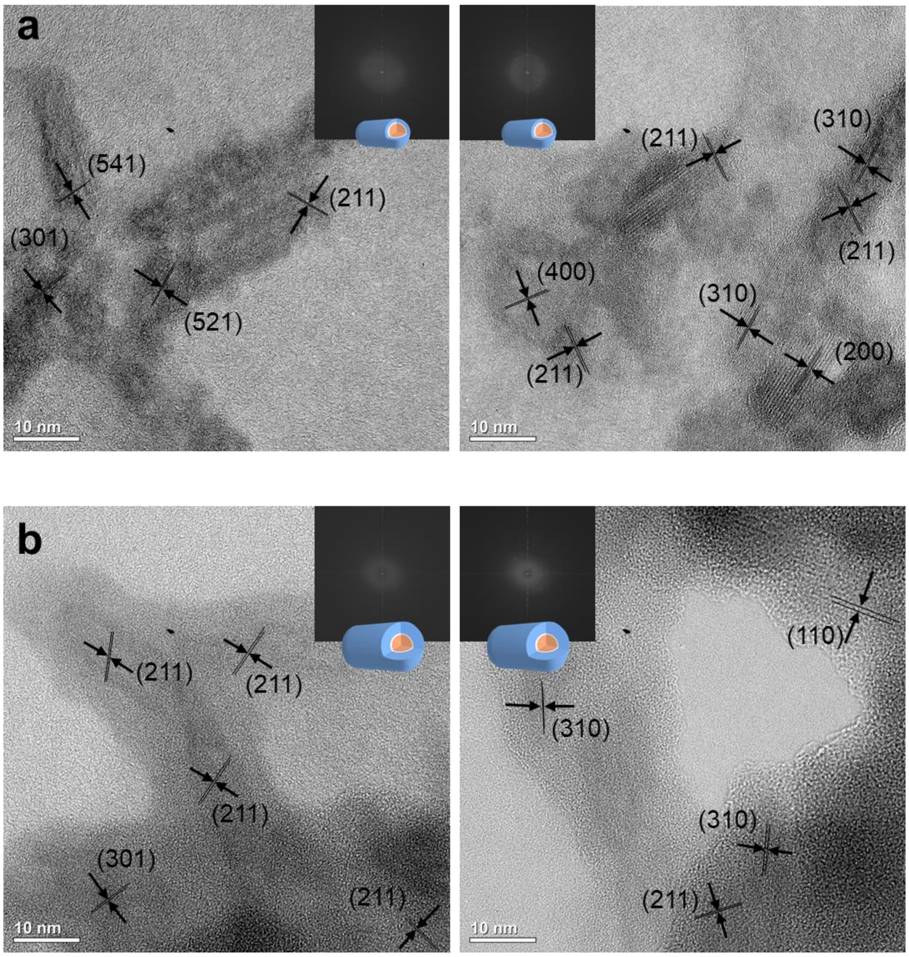

Supplementary Figure 3.4. Additional HR-TEM images of the silica-functionalized akaganeite precursors with a) 2.6 nm (S3-2) and b) 5.6 nm (S3-8) layer thickness.

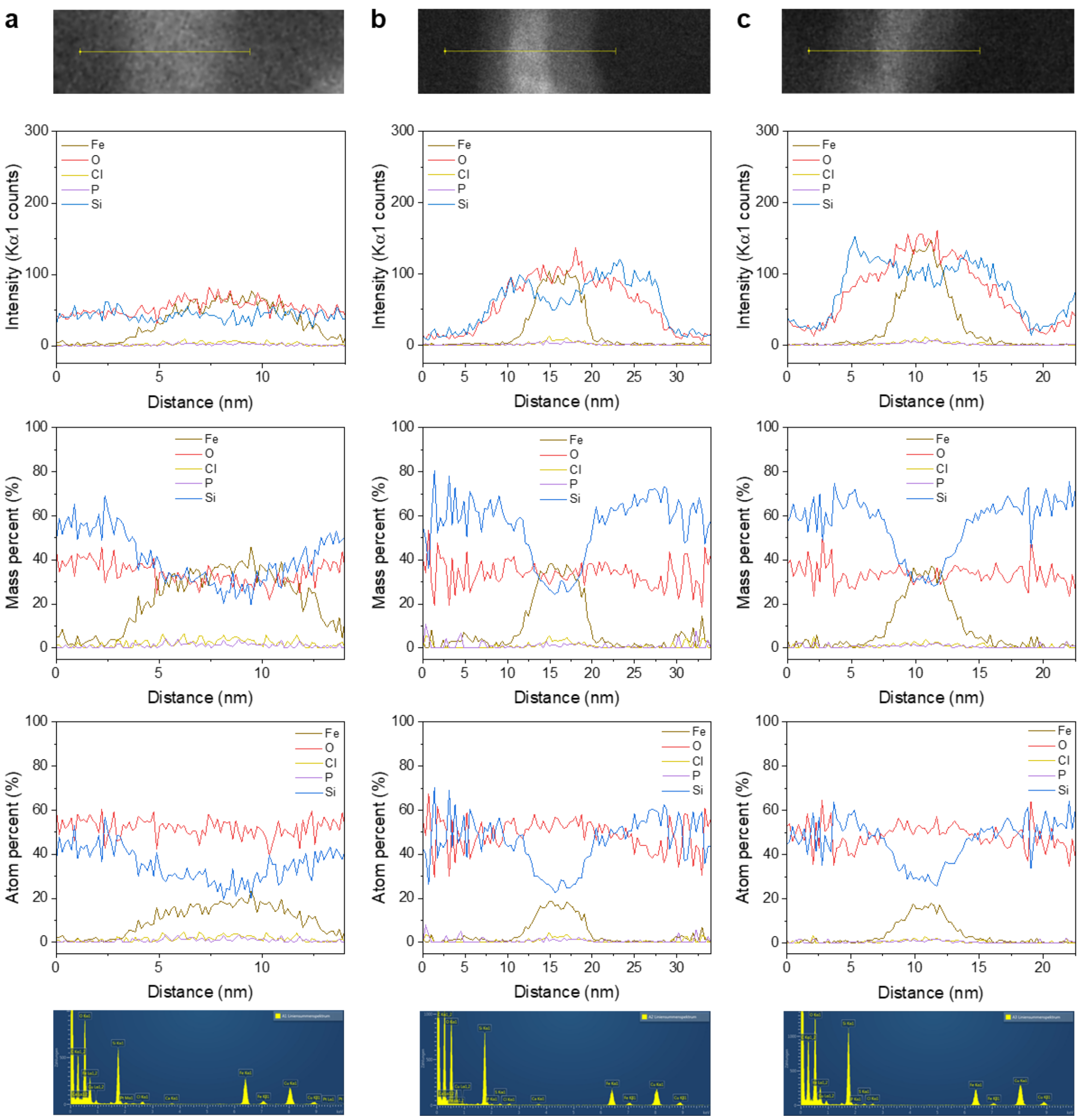

Supplementary Figure 3.5. Line scan profiles and line sum spectra of sample S3-8 (d_{SiO2} = 5.6 nm). a-c) Three different line scan profiles demonstrate the enhancement of the silica signal on the edges of the akaganeite particles (Fe and Cl signal). The silica shell can be measured using the intensity illustration of **b** and **c**, confirming the thickness of around 5 to 6 nm from TEM analysis. The Si/Fe ratio is (3.8 ± 1.5), which results in approximately a thirteenfold increase compared to S3-2.

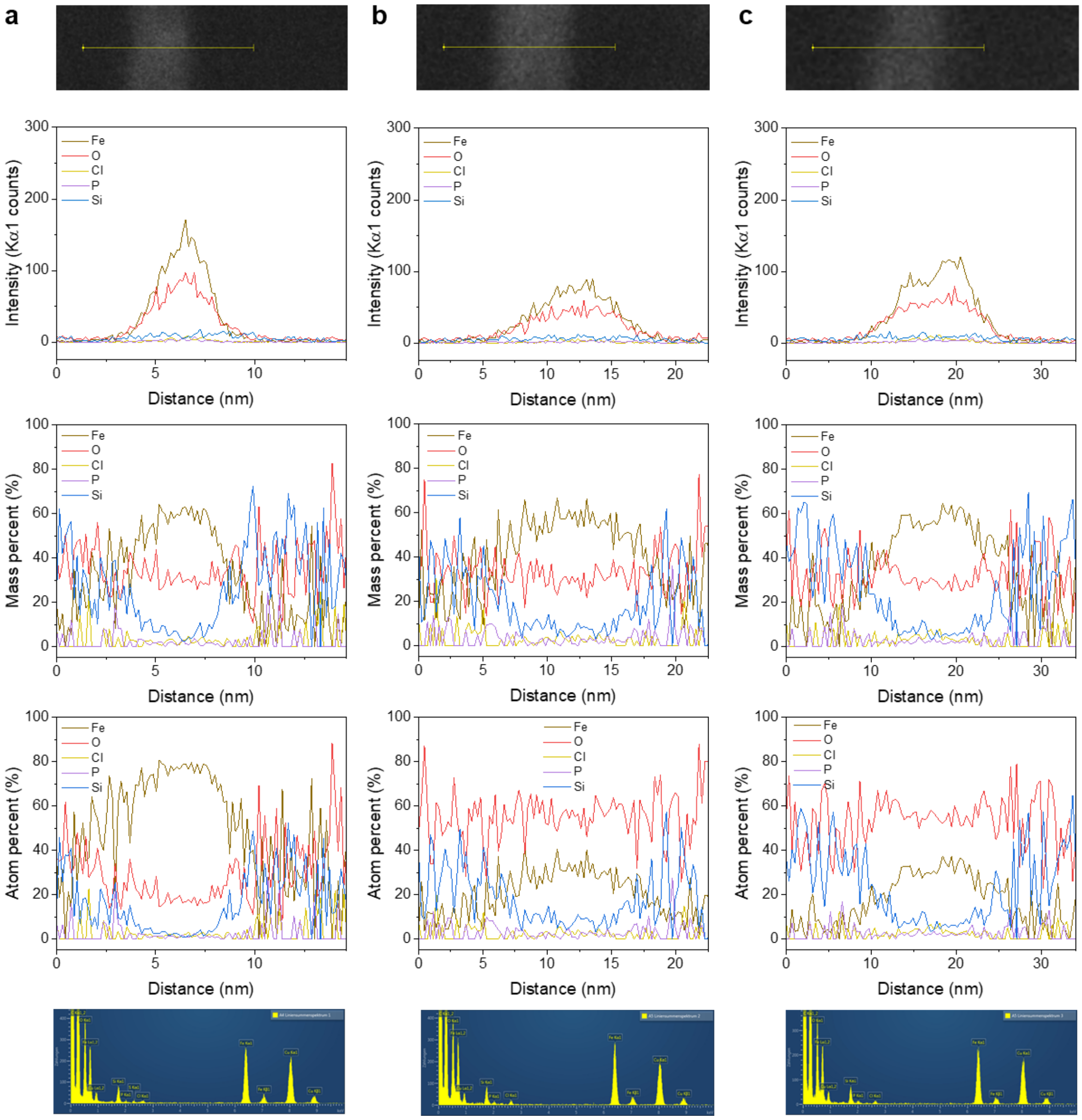

Supplementary Figure 3.6. Line scan profiles and line sum spectra of sample S3-2 (d_{SiO2} = 2.6 nm). a-c) Three different line scan profiles demonstrate the enhancement of the silica signal on the edges of the akaganeite particles (Fe and Cl signal). The silica shell can be measured using the intensity illustration of **b** and **c**, confirming the thickness of around 2 to 3 nm from TEM analysis. The Si/Fe ratio is (0.28 ± 0.003).

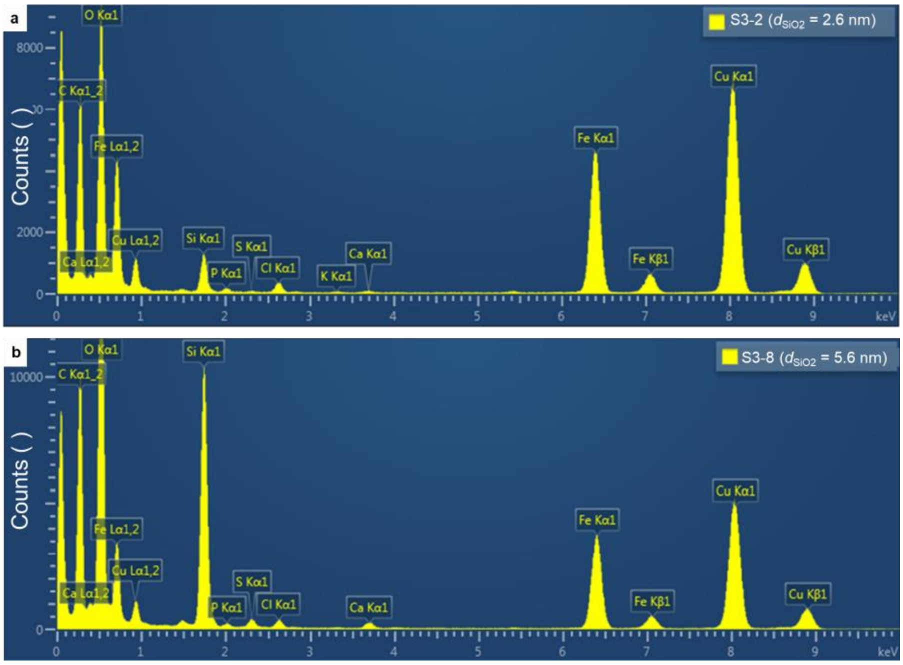

Supplementary Figure 3.7. Sum spectra of two samples with different silica thicknesses. On average, a sum distribution of Fe, O, Cl, P, and Si for **a)** sample S3-2 with 19.8, 73.5, 1.2, 0.5, and 5.0 atom% and **b)** sample S3-8 with 7.6, 71.1, 0.6, 0.2, and 20.5 atom% is obtained, respectively. The Si/Fe ratio increases from 0.28 to 2.7 as the silica shell thickness increases.

Supplementary Note 3.4. Silica Porosity

Additionally, the HR-TEM analysis allows for a cautious interpretation of the porosity of the silica shells, which lack crystal planes and are therefore more likely to exhibit an amorphous or microporous (pore diameter of <2 nm) structure. Comparable porosities have been investigated in the literature, as explained later, and determined using BET analysis for similarly synthesized silica shells produced *via* the Stöber process. Such a structure could dissolve under alkaline conditions, with the dissolution time depending on the thickness of the silica layer.[17]

An examination of the porosity, pore sizes, and specific surface areas of the samples using gas adsorption was not conducted in this work, as the silica-functionalized particles are present in aqueous solution after the washing processes. The specific surface area of the silica-functionalized particles would change during the drying process used to produce the solid measurement sample due to the agglomeration of the individual particles through a condensation reaction. It may be possible to determine pore volume or pore diameter. Still, these would not only be based on existing pores in the silica layer, but could also result from voids between individual particles within the agglomerates. Grinding the dried sample afterward would lead to a significant alteration. Therefore, no meaningful data are expected for the specific surface area and porosity. It should also be noted that (especially N_2) adsorption measurements at 77 K typically yield 20–30 times lower specific surface values than other methods (*e.g.*, pycnometry, acid-base titration, liquid-phase adsorption, SAXS) at higher temperatures, as noted by Szekeres *et al.*[18] Furthermore, specific surface areas calculated *via* BET models tend to be slightly higher than geometric surface areas, indicating the presence of non-porous structures. In any case, statements derived from different measurement methods are associated with difficulties and should not be overlooked. Therefore, comparative literature values for the porosity, pore sizes, and specific surface areas were added based on our Stöber synthesis as follows.

The Stöber process itself is well understood, with reviews on the nucleation, growth process, and their microstructure.[19,20] Silica spheres synthesized *via* the Stöber process are generally described as nonporous. However, synthesis parameters can induce a certain fraction of microporosity, as shown in the literature.[21,22] Li *et al.* investigated the pore characteristics using N_2 adsorption measurements, finding specific surface areas ranging from 11.3 to 309.7 $m^2\ g^{-1}$. In summary, a short reaction time, washing steps with water, and vacuum drying of the structures result in high specific surface areas. A comparable sample to our work, where the Stöber-synthesized silica spheres were first washed twice with ethanol and then three times with water, achieving a surface area of 153.0 $m^2\ g^{-1}$. The pore diameters were 1.2 nm, the total pore volume 0.177 $cm^3\ g^{-1}$, and the micropore volume 0.047 $cm^3\ g^{-1}$.

Bazuła *et al.* created microporous structures with significantly increased specific surface areas by re-immersing Stöber-synthesized silica spheres in ethanol, resulting in the re-esterification of silanol groups.[23] Additional washing with water removes alkoxy groups via hydrolysis, resulting in pure microporous spheres with pore diameters of 2 – 4 nm. N_2 adsorption measurements showed low pore volumes of 0.01 $cm^3\ g^{-1}$ and specific surface areas of 9 $m^2\ g^{-1}$ for untreated samples (washed only three times with water), which increased to 0.20 $cm^3\ g^{-1}$ and 385 $m^2\ g^{-1}$, respectively, after further washing steps. This treatment can lead to porosities of up to 31 vol.%. It is important to note that storing these samples in water for three days reduces micropore volume without affecting the total pore volume, which now includes mesopores with diameters of 4 – 8 nm. This rationale underpins our decision to utilize freshly synthesized silica precursors in the hydrothermal step and to avoid storing them.

Porosity measurement is more frequently performed *via* argon adsorption, which provides more accurate results than nitrogen adsorption at 77 K due to the smaller kinetic diameter and lack of quadrupole moment of argon. A stepwise increase in adsorption isotherms indicates fill levels of micropores and small mesopores. The absence of hysteresis between adsorption and desorption

isotherms suggests a lack of larger mesopores that would occur through capillary condensation. A surfactant-controlled silica synthesis producing spheres and fibers results in mesopores with an average diameter of 2.8 to 3.5 nm.[24] The specific surface areas are 730 and 980 m^2 g^{-1} for spheres and fibers, respectively. The structure of the hexagonally ordered mesopores is confirmed by XRD, showing (100), (110), and (200) reflections at 2.5°, 4.3°, and 4.9°, respectively. In contrast, spheres only show (100) reflections, indicating a less ordered pore system. These reflections are also observed in the XRD patterns of our silica-functionalized precursor, suggesting a lower degree of order.

Supplementary Section 4. Size-Dependent Synthesis and Arrangement of Cobalt Ferrite Chains: Overview

Supplementary Note 4.1. Size Calculation from XRD Data.

The crystallite size was obtained by two different calculations using a) Debye-Scherrer (DS) plots or b) Williamson-Hall (WH) plots.

The crystallite size d_{DS} was determined through the following mathematical relationship (Supplementary Equation 4.1) using a Gauss fit at reflex (311) of cobalt ferrite, where β is the line broadening at half of the maximum intensity (FWHM), and k is a dimensionless shape factor fixed at 0.9 for cubic crystallites:

$$d_{DS} = \frac{\lambda \cdot k}{\beta \cdot \cos(\theta)} \quad 4.1$$

The analysis of the crystallite size across all reflections through WH plots enables a more precise determination. By linear fitting of WH plots, the crystallite size d_{WH} is obtained by ordinate intercept according to Supplementary Equation 4.2:

$$\beta \cdot \cos(\theta) = 4 \cdot \varepsilon \cdot \sin(\theta) + \frac{\lambda \cdot k}{d_{WH}} \quad 4.2$$

Supplementary Note 4.2. Cobalt Content Calculation from F-AAS Data.

The cobalt-to-iron ratio κ was calculated for all samples using Equation 10 to 12 based on Supplementary Equation 4.3 with

$$n_{CF}(Co) + n_{CF}(Fe) = 3. \quad 4.3$$

The ratio κ further indicates that the following stoichiometry for iron and cobalt is present:

$$x_{CF}(Co) = \frac{3\kappa}{1+\kappa} \text{ and} \quad 4.4$$

$$x_{CF}(Fe) = 3 - x_{CF}(Co). \quad 4.5$$

The dispersity values obtained from the TEM determinations are calculated using the Supplementary Equation 4.6.

$$\mathrm{PDI} = \left(\frac{\sigma}{\mu}\right)^2 \quad 4.6$$

Supplementary Table 4.1. Characterization of the cobalt-doped ferrite (CF) samples. CF samples were synthesized with different precursors (akaganeite (A), PAA-modified akaganeite (P), or silica-functionalized akaganeite (S)) and characterized regarding their diameter obtained via TEM and XRD, their cobalt-to-iron-ratio, and cobalt content obtained *via* F-AAS. Mean values (M) are calculated.

**Sample**	**Silica layer thickness**		**Cobalt-to-iron ratio**		**Stoichiometric cobalt coefficient**		**Diameter**						**Angle at (311) reflex**	
	d_{SiO2}	σ_{SiO2}	κ	σ_{κ}	x_{Co}	σ_{Co}	d_{TEM}	σ_{TEM}	d_{DS}	σ_{DS}	d_{WH}	σ_{WH}	2θ	$\sigma_{2\theta}$
	**(nm)**	**(nm)**	**( )**	**( )**	**( )**	**( )**	**(nm)**	**(nm)**	**(nm)**	**(nm)**	**(nm)**	**(nm)**	**(°)**	**(°)**
**CF-A1**	0.0	± 0.0	0.158	± 0.002	0.41	± 0.00	25.3	± 7.5	19.0	± 0.2	21.0	± 1.1	35.382	± 0.02
**CF-P1**	0.0	± 0.0	0.165	± 0.001	0.42	± 0.00	16.0	± 4.9	17.6	± 0.2	19.4	± 0.5	35.441	± 0.02
**CF-S1-1**	2.5	± 3.2	0.180	± 0.000	0.46	± 0.00	26.9	± 12.3	25.9	± 0.4	34.6	± 0.9	35.435	± 0.02
**CF-S1-2**	2.5	± 3.2	0.196	± 0.001	0.49	± 0.00	43.2	± 12.6	28.0	± 0.4	40.6	± 2.1	35.431	± 0.02
CF-S1-M	*2.5*	*± 3.2*	*0.188*	*± 0.008*	*0.47*	*± 0.02*	*35.1*	*± 8.2*	*27.0*	*± 1.1*	*37.6*	*± 3.0*	*35.433*	*± 0.02*
**CF-A2**	0.0	± 0.0	0.171	± 0.002	0.44	± 0.01	20.0	± 7.0	24.2	± 0.4	29.7	± 1.7	35.355	± 0.02
**CF-P2**	0.0	± 0.0	0.159	± 0.001	0.41	± 0.00	14.0	± 3.0	17.8	± 0.2	19.3	± 1.4	35.430	± 0.02
**CF-S2-1**	2.5	± 3.2	0.182	± 0.005	0.46	± 0.01	34.8	± 11.1	26.1	± 0.4	38.1	± 4.7	35.428	± 0.02
**CF-S2-2**	2.5	± 3.2	0.194	± 0.000	0.49	± 0.00	46.9	± 14.9	28.5	± 0.4	45.6	± 1.9	35.423	± 0.02
CF-S2-M	*2.5*	*± 3.2*	*0.188*	*± 0.006*	*0.48*	*± 0.01*	*40.9*	*± 6.1*	*27.3*	*± 1.2*	*41.9*	*± 3.8*	*35.425*	*± 0.02*
**CF-A3**	0.0	± 0.0	0.163	± 0.002	0.42	± 0.00	25.7	± 6.1	26.3	± 0.5	35.5	± 3.0	35.579	± 0.03
**CF-P3-1**	0.0	± 0.0	0.180	± 0.002	0.46	± 0.01	18.7	± 5.9	16.9	± 0.2	19.6	± 1.2	35.503	± 0.02
**CF-P3-2**	0.0	± 0.0	0.170	± 0.000	0.44	± 0.00	17.9	± 5.5	16.7	± 0.3	19.5	± 0.7	35.493	± 0.04
CF-P3-M	*0.0*	*± 0.0*	*0.175*	*± 0.005*	*0.45*	*± 0.01*	*18.3*	*± 0.4*	*16.8*	*± 0.1*	*19.5*	*± 0.1*	*35.498*	*± 0.05*
**CF-S3-1**	2.2	± 1.5	0.171	± 0.003	0.44	± 0.01	22.0	± 10.8	19.0	± 0.2	20.9	± 1.3	35.472	± 0.02
**CF-S3-3**	3.4	± 1.7	0.171	± 0.003	0.44	± 0.01	19.9	± 7.1	18.4	± 0.2	21.1	± 3.0	35.439	± 0.02
**CF-S3-4**	3.7	± 1.7	0.157	± 0.005	0.41	± 0.01	22.5	± 6.7	20.6	± 0.4	24.3	± 1.7	35.344	± 0.03
**CF-S3-5**	4.0	± 1.6	0.176	± 0.001	0.45	± 0.00	18.5	± 3.7	19.1	± 0.3	21.4	± 1.5	35.569	± 0.02
**CF-S3-6***	5.3	± 2.1	0.231	± 0.003	0.56	± 0.00	45.5	± 15.6	24.7	± 0.5	42.2	± 8.5	35.481	± 0.03
**CF-S3-8**	5.6	± 2.4	0.185	± 0.001	0.47	± 0.00	34.2	± 12.2	26.1	± 0.4	36.0	± 4.9	35.452	± 0.02
**CF-S3-9**	6.8	± 1.9	0.195	± 0.002	0.49	± 0.00	40.1	± 14.2	29.3	± 0.5	48.5	± 7.9	35.436	± 0.02

*Sample CF-S3-6 shows a significantly higher cobalt content ($x = 0.56$, $\kappa = 0.23$) after the hydrothermal step, so we suggest that this sample was prepared with the wrong weight-in of the cobalt salt.

Supplementary Table 4.2. Dispersity of the cobalt-doped ferrite (CF) samples using the mean values and size deviations of the TEM measurements.

Sample	Silica layer thickness		Diameter		Dispersity
	d_{SiO2}	σ_{SiO2}	d_{TEM}	σ_{TEM}	*PDI*
	(nm)	(nm)	(nm)	(nm)	( )
**CF-A1**	0.0	± 0.0	25.3	± 7.5	0.09
**CF-P1**	0.0	± 0.0	16.0	± 4.9	0.09
**CF-S1-1**	2.5	± 3.2	26.9	± 12.3	0.21
**CF-S1-2**	2.5	± 3.2	43.2	± 12.6	0.09
CF-S1-M	*2.5*	*± 3.2*	*35.1*	*± 8.2*	*0.15*
**CF-A2**	0.0	± 0.0	20.0	± 7.0	0.12
**CF-P2**	0.0	± 0.0	14.0	± 3.0	0.04
**CF-S2-1**	2.5	± 3.2	34.8	± 11.1	0.10
**CF-S2-2**	2.5	± 3.2	46.9	± 14.9	0.10
CF-S2-M	*2.5*	*± 3.2*	*40.9*	*± 6.1*	*0.10*
**CF-A3**	0.0	± 0.0	25.7	± 6.1	0.06
**CF-P3-1**	0.0	± 0.0	18.7	± 5.9	0.10
**CF-P3-2**	0.0	± 0.0	17.9	± 5.5	0.10
CF-P3-M	*0.0*	*± 0.0*	*18.3*	*± 0.4*	*0.10*
**CF-S3-1**	2.2	± 1.5	22.0	± 10.8	0.24
**CF-S3-3**	3.4	± 1.7	19.9	± 7.1	0.13
**CF-S3-4**	3.7	± 1.7	22.5	± 6.7	0.09
**CF-S3-5**	4.0	± 1.6	18.5	± 3.7	0.04
**CF-S3-6***	5.3	± 2.1	45.5	± 15.6	0.12
**CF-S3-8**	5.6	± 2.4	34.2	± 12.2	0.13
**CF-S3-9**	6.8	± 1.9	40.1	± 14.2	0.13

Supplementary Section 5. Size-Dependent Synthesis and Arrangement of Cobalt Ferrite Chains: Temperature Effect

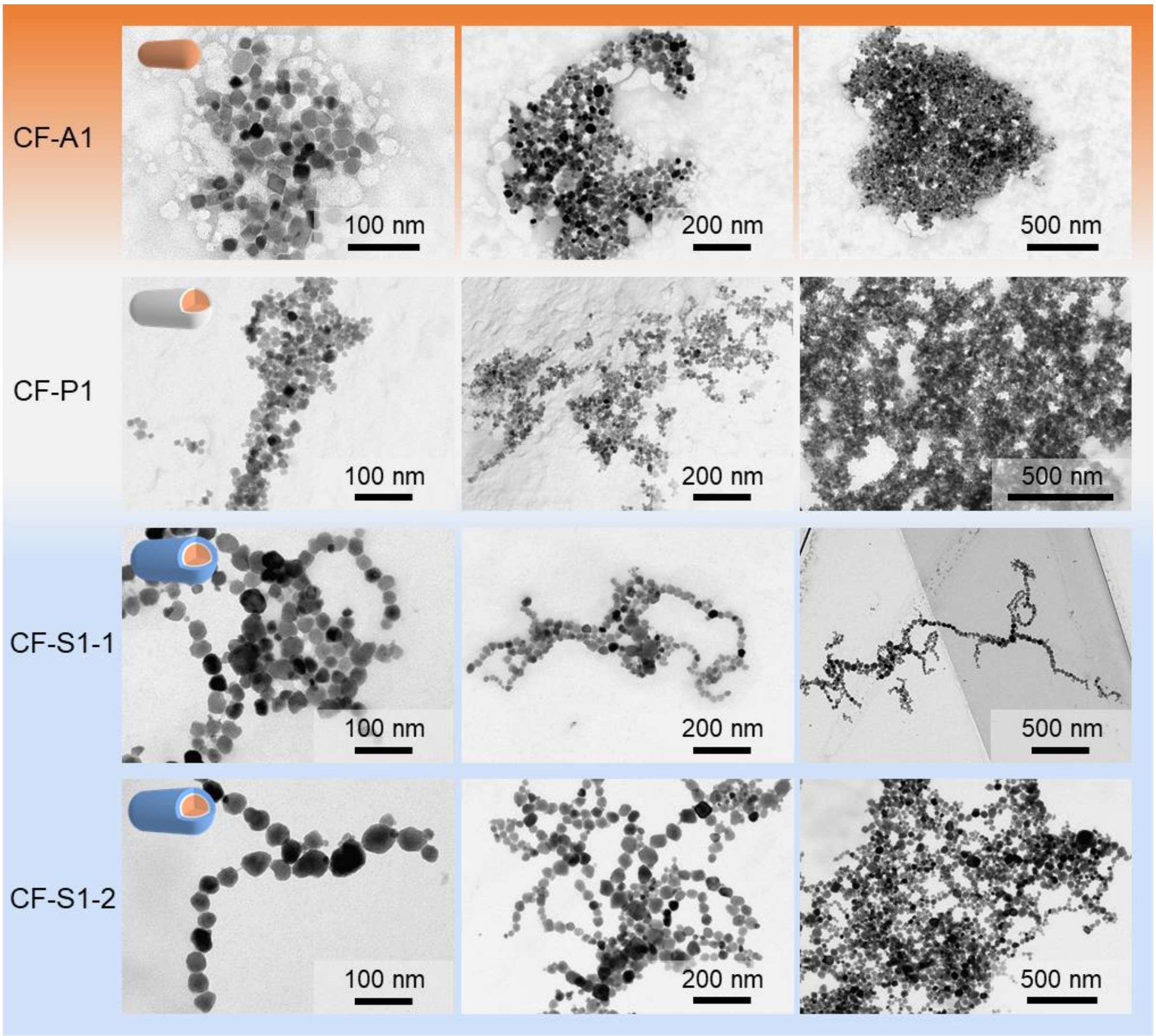

Supplementary Figure 5.1. TEM images in different magnifications of cobalt ferrite nanoparticles of series S1. The samples were synthesized with the bare precursor akaganeite, the PAA-modified precursor, or the precursor aka@0.12PAA@2.5SiO2 with a shell thickness of 2.5 nm in a hydrothermal step with a filling volume of 100% at a maximum temperature of 160 °C.

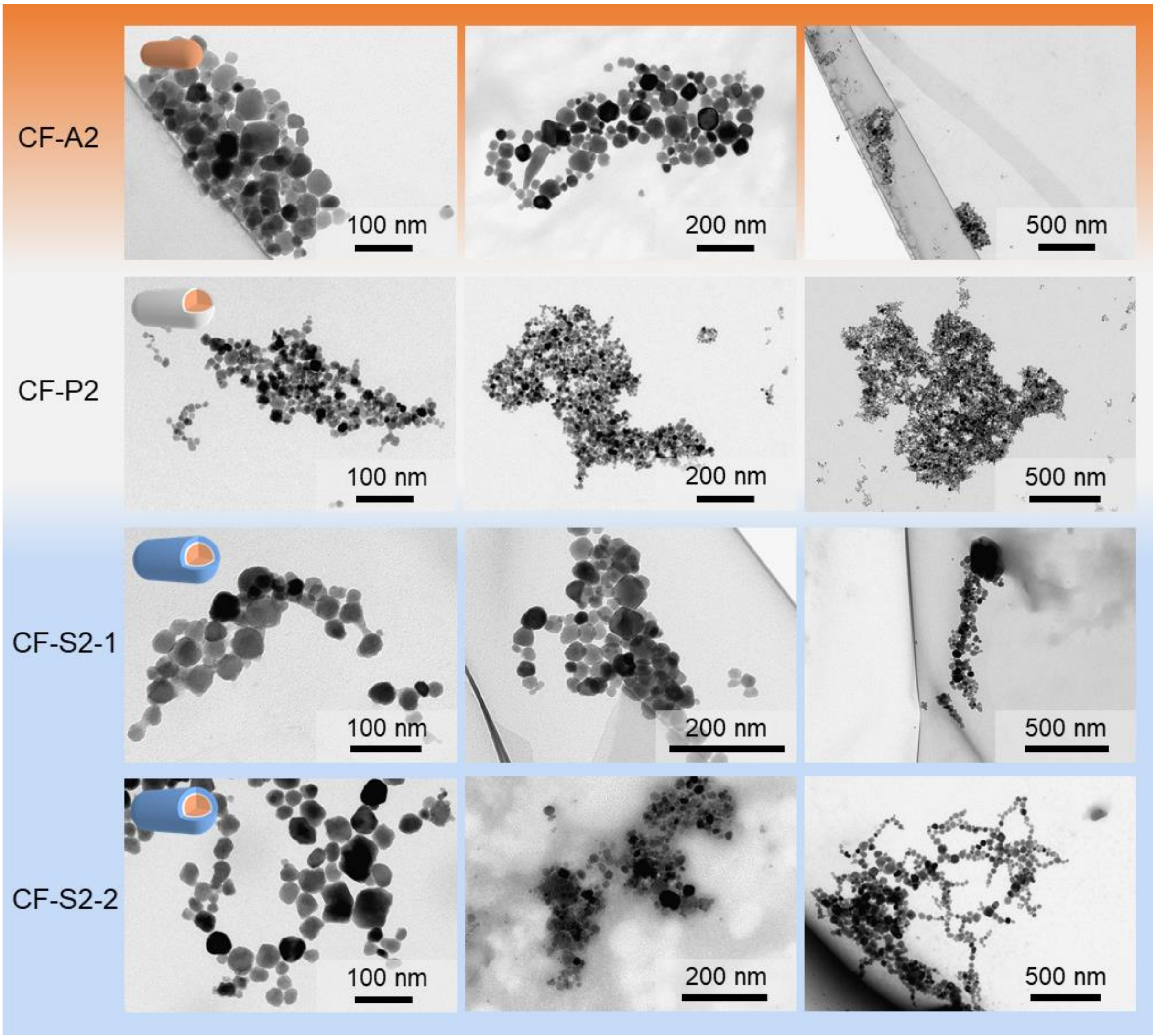

Supplementary Figure 5.2. TEM images in different magnifications of CF nanoparticles of series S2. The samples were synthesized with the bare precursor akaganeite, the PAA-modified precursor, or the precursor aka@0.12PAA@2.5SiO2 with a shell thickness of 2.5 nm in a hydrothermal step with a filling volume of 100% at a maximum temperature of 190 °C.

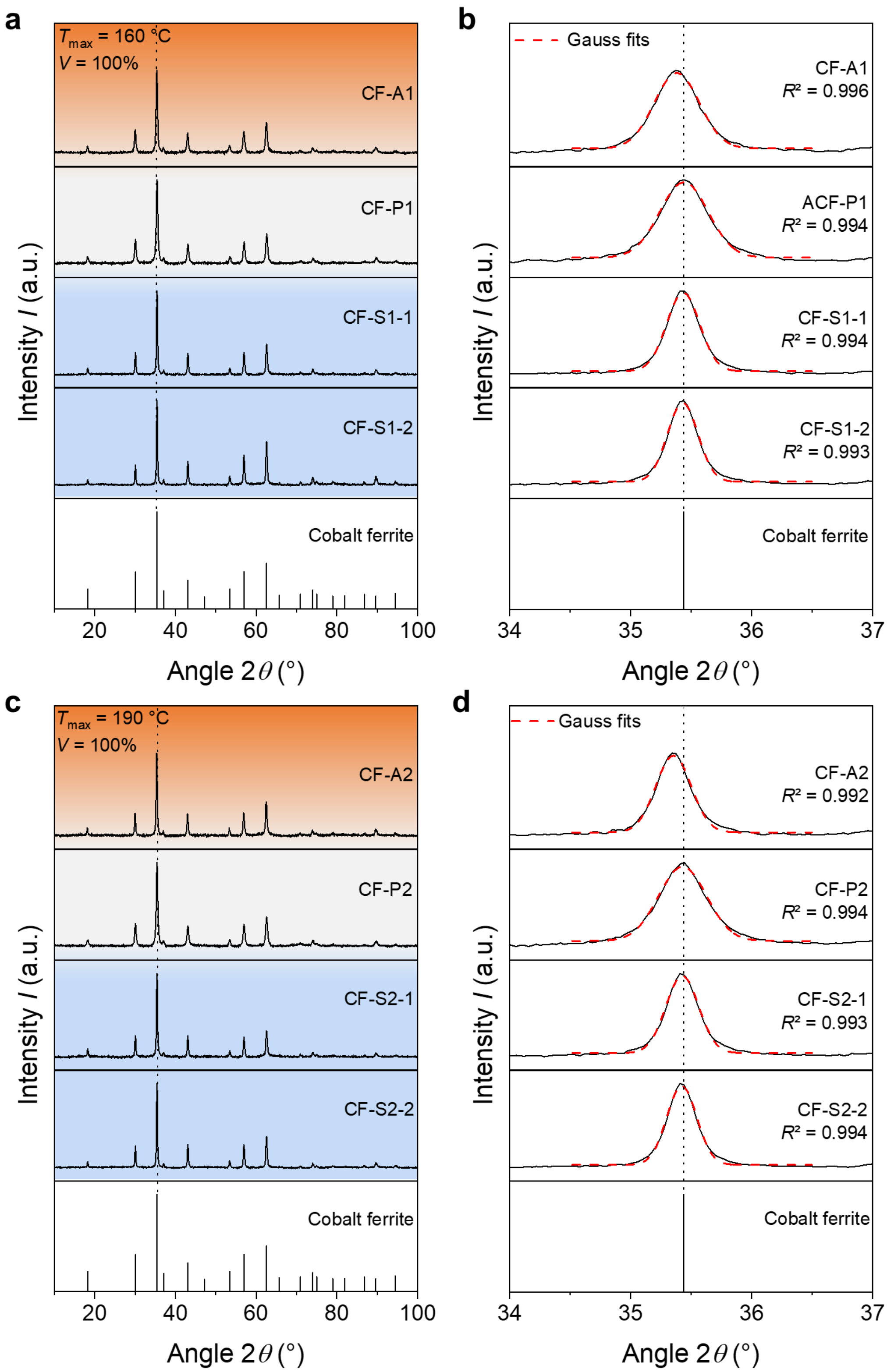

Supplementary Figure 5.3. Composition of the cobalt ferrite nanoparticles synthesized at different temperatures and with a filling volume of 100%. The diffraction patterns are presented with the background intensity eliminated through a second derivative fitting to zero. The observed peaks of the diffraction patterns match the cobalt ferrite phase (JCPS No. 00-022-1086) in very good agreement regarding the intensities and angle values for **a**) T_{max} = 160 °C and **c**) T_{max} = 190 °C. The inlet of the diffraction patterns at the reflex (311) are used for the calculation of the crystallite size for **b**) T_{max} = 160 °C and **d**) T_{max} = 190 °C. Furthermore, the samples synthesized with the pure akaganeite precursors show a slight shift to lower angles. With a reactor filling volume of 100%, the reflex (311) of the magnetic particles observed with blank akaganeite nanorods is shifted to smaller angles meaning a smaller cobalt amount is included in the lattice and the diffraction pattern shifted to magnetite reflexes. By using modified nanorods with polymer or silica shell, pure cobalt ferrite reflexes can be obtained.

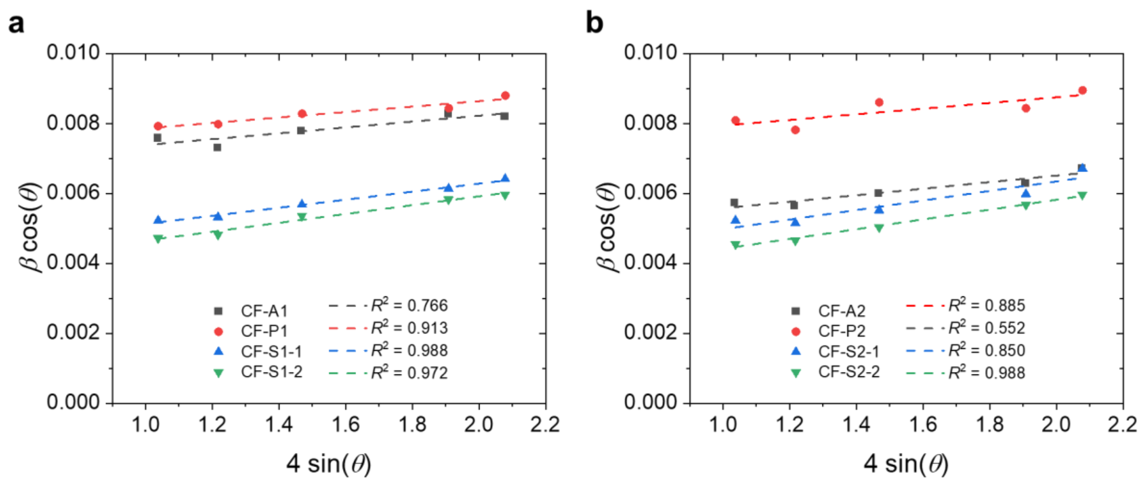

Supplementary Figure 5.4. Williamson-Hall plots of the CF samples synthesized with a filling volume of 100 %. WH plots and corresponding linear fits with adjusted R^2-value for **a**) 160 °C and **b**) 190 °C.

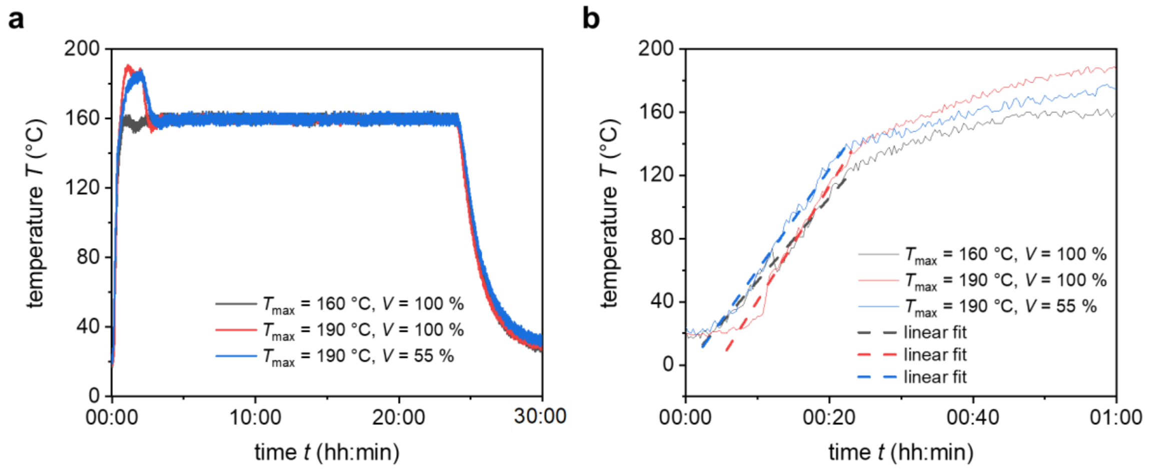

Supplementary Figure 5.5. Heating rates of the hydrothermal reaction. **a**) Different temperature curves are observed using different heating programs with a maximum temperature set to 160 °C or 190 °C for 90 minutes. **b**) The heating rates of 6.6 K/min (red), 5.6 K/min (blue), and 5.0 K/min (black) up to 20 minutes reaction time can be adjusted by using different filling volumes and maximum temperature sets. After this time, the heating rates shrink due to the built-up and constant pressure of approximately 5 bar in the reactor.

Supplementary Section 6. Size-Dependent Synthesis and Arrangement of Cobalt Ferrite Chains: Silica Shell Thickness

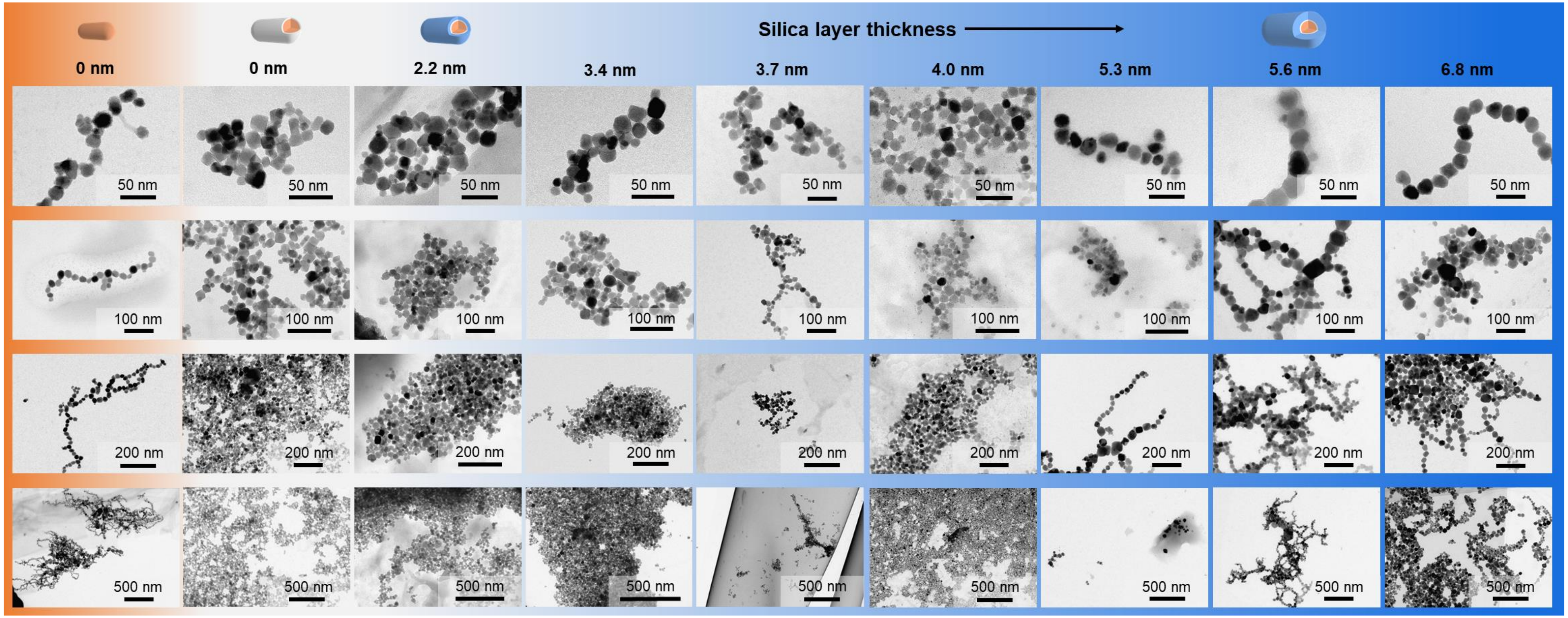

Supplementary Figure 6.1. TEM images in different magnifications of CF nanoparticles of series S3. The samples were synthesized with the bare precursor akaganeite, the PAA-modified precursor, or the precursor aka@0.12PAA@SiO2 with a shell thickness of d_{SiO2} (nm) in a hydrothermal step with a filling volume of 55% at a maximum temperature setting of 190 °C. An overview of the particle shape, size, and arrangement is shown in TEM images observed with different magnifications (Supplementary Figure 6.1). The diameter d_{TEM} was observed by measuring 150 particles and calculating the mean average and sigma value.

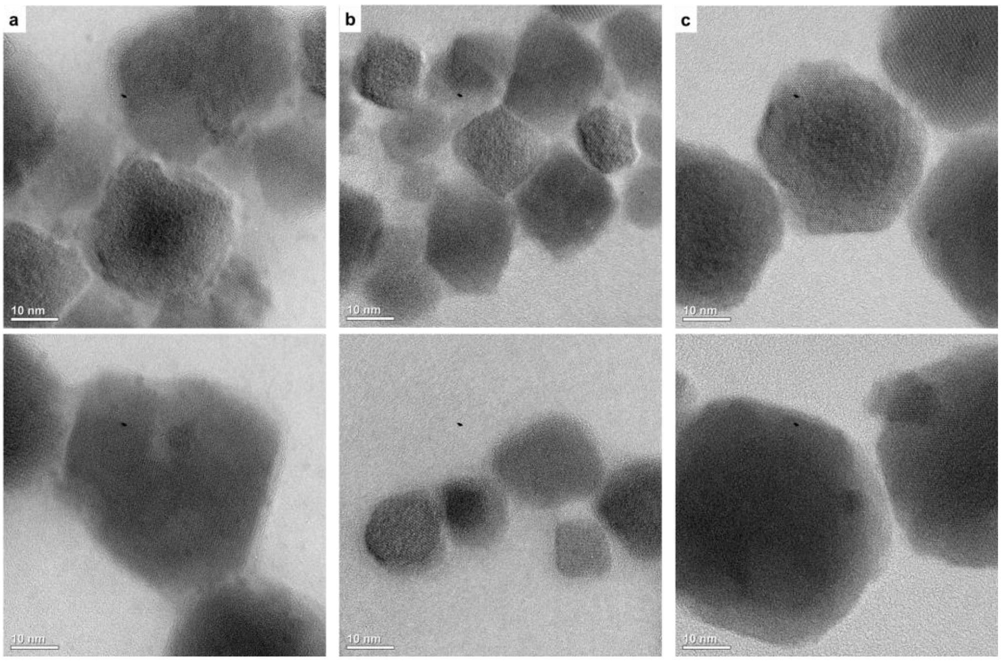

Supplementary Figure 6.2. HR-TEM images of the CF nanoparticles synthesized at 190 °C with a filling volume of 55% using different precursors. The morphology differs slightly between **a**) CF-A3, **b**) CF-P3-1, and **c**) CF-S3-8.

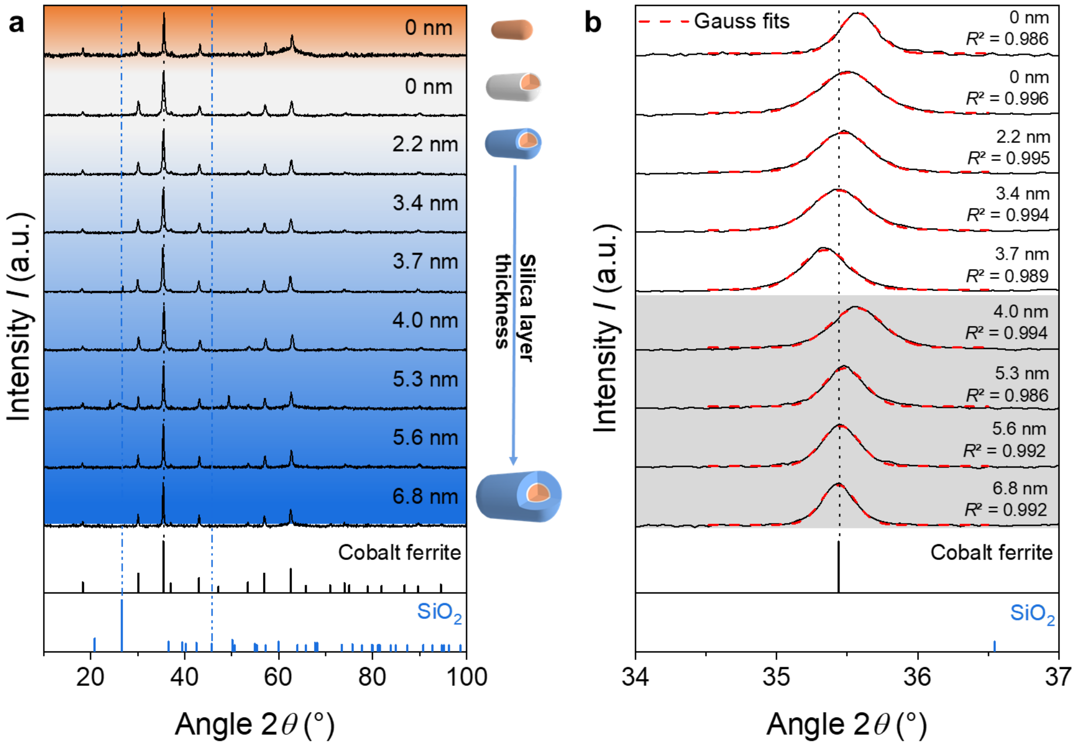

Supplementary Figure 6.3. Composition of the CF nanoparticles synthesized at 190 °C with a filling volume of 55 % using precursors with different silica layer thicknesses. **a**) The observed peaks of the diffraction patterns match the cobalt ferrite phase (JCPS No. 00-022-1086) in good agreement regarding the intensities and angle values for all approaches. **b**) The inlet of the diffraction patterns at the reflex (311) are used for the calculation of the crystallite size. It can be observed that there is the same shift behavior to smaller angles in dependency on the silica thicknesses from 0 to 3.4 nm and above 4 nm. The 2θ angle of the (311) reflex shows a slight shift to smaller angles. The diameter d_{DS} of each sample was determined by fitting the (311) reflex of each diffraction pattern by a Gauss curve (Supplementary Figure 6.2). The observed FWHM and 2θ angle at maximum were used to calculate d_{DS} using the Debye-Scherrer equation (Equation 6), as well as the σ values *via* differential error analysis.

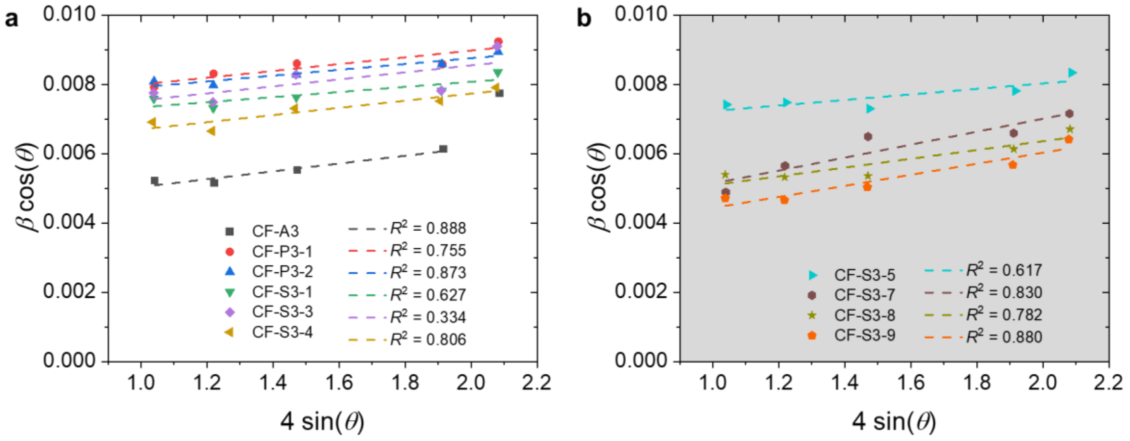

Supplementary Figure 6.4. Williamson-Hall plots of the CF samples synthesized at 190 °C with a filling volume of 55 % using precursors with different silica layer thicknesses. A more precise determination of the diameter d_{WH} was obtained through the application of the Williamson-Hall plot (Equation 7) across all reflections (Supplementary Figure 6.3). WH plots and corresponding linear fits with adjusted R^2-value of samples synthesized with a silica thickness **a**) below 4 nm and **b**) above 4 nm.

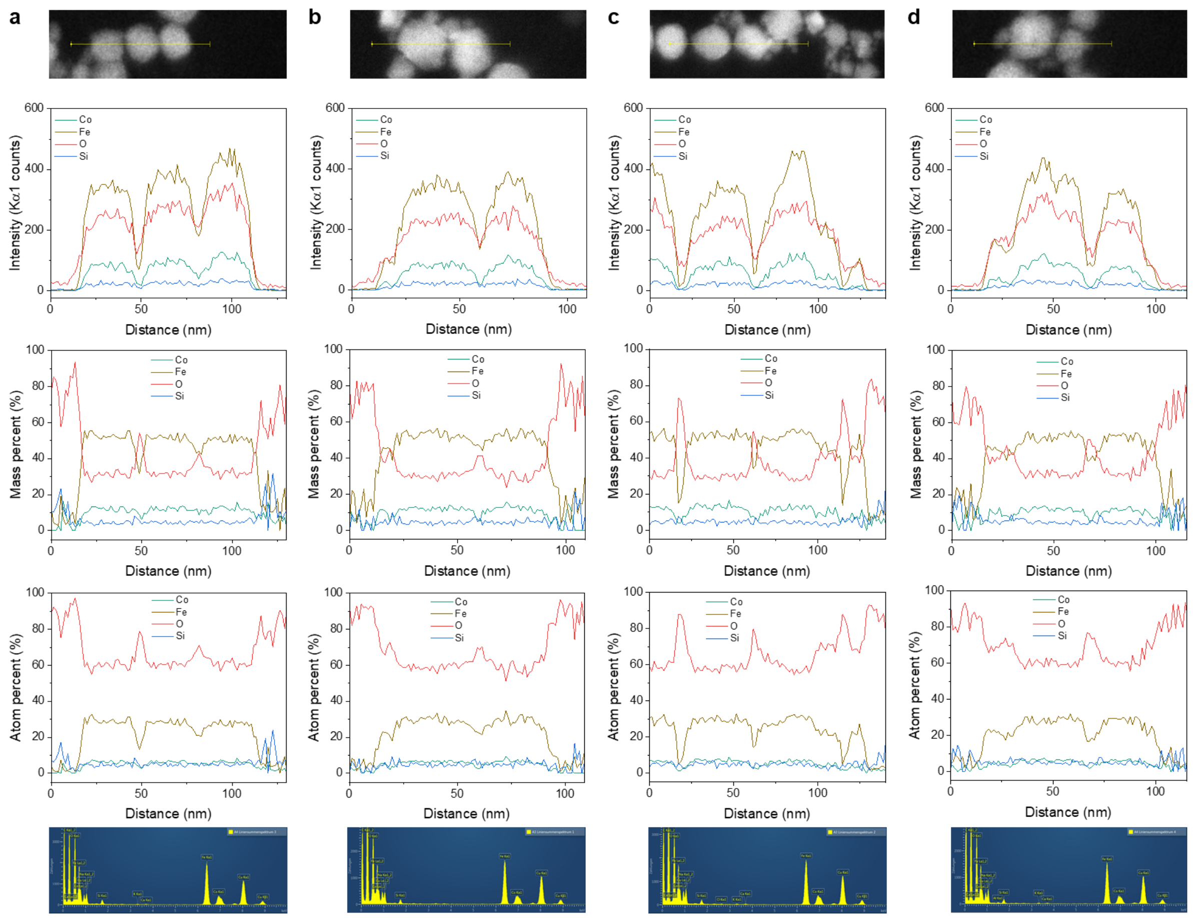

Supplementary Figure 6.5. Line scan profiles and line sum spectra of sample CF-A3. a-d) Four different areas show a similar line sum distribution of Co, Fe, O, and Si with (4.8 ± 0.3), (23.1 ± 0.6), (69.9 ± 0.8), and (2.2 ± 0.1) atom%, on average. However, the distribution includes only the intensities of Si, Co, Fe, and O, which were normalized to 100 mass% and 100 atom%. Therefore, the absolute values should be lower when considering all elements, as other components are not included in this normalization. The Co/Fe ratio is (0.210 ± 0.009).

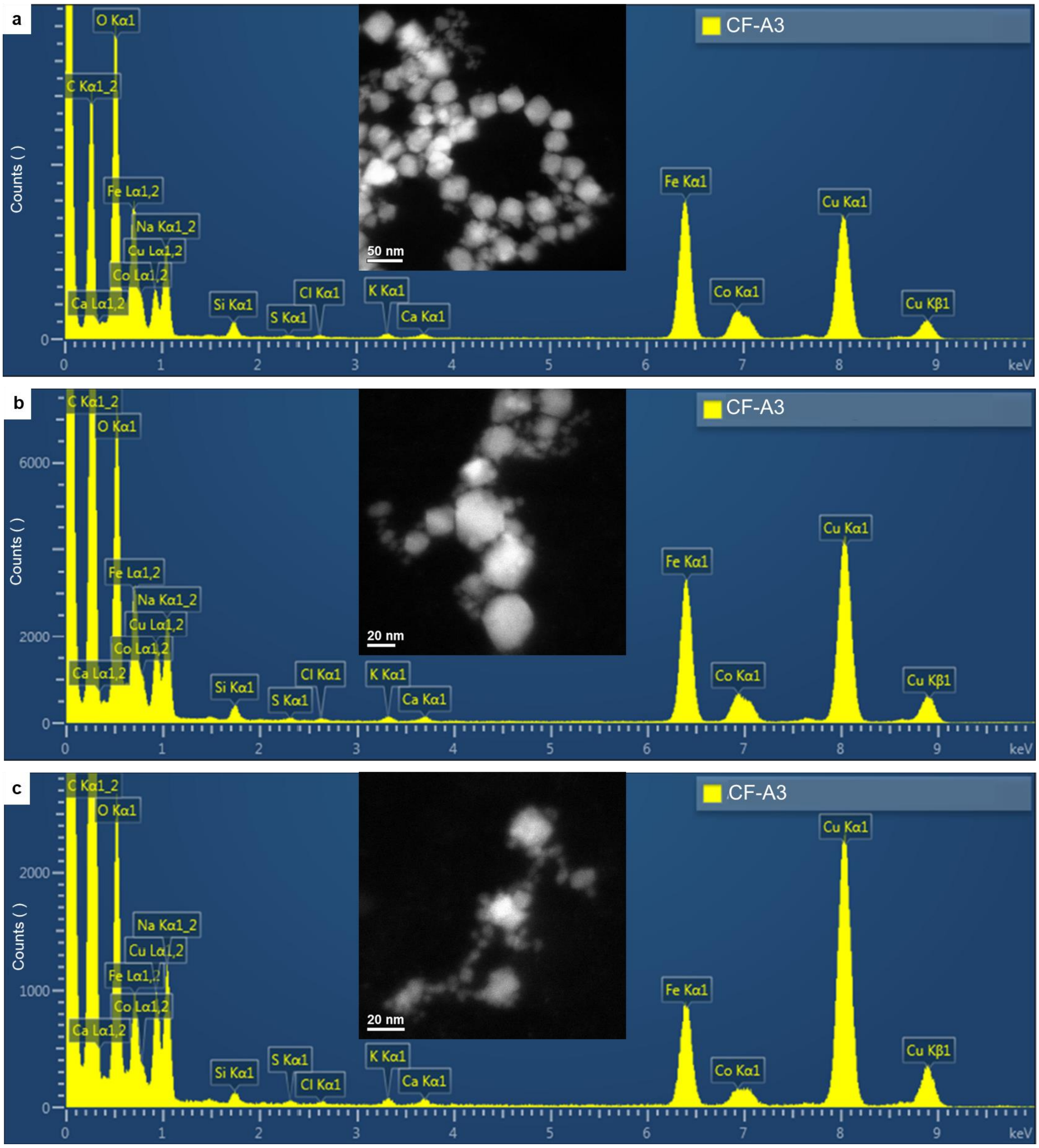

Supplementary Figure 6.6. Map sum spectra of sample CF-A3. Three different areas show a similar sum distribution of Co, Fe, O, and Si with (3.3 ± 0.8), (17.4 ± 2.1), (77.6 ± 3.0), and (1.8 ± 0.2) atom%, on average. The Co/Fe ratio is (0.187 ± 0.025).

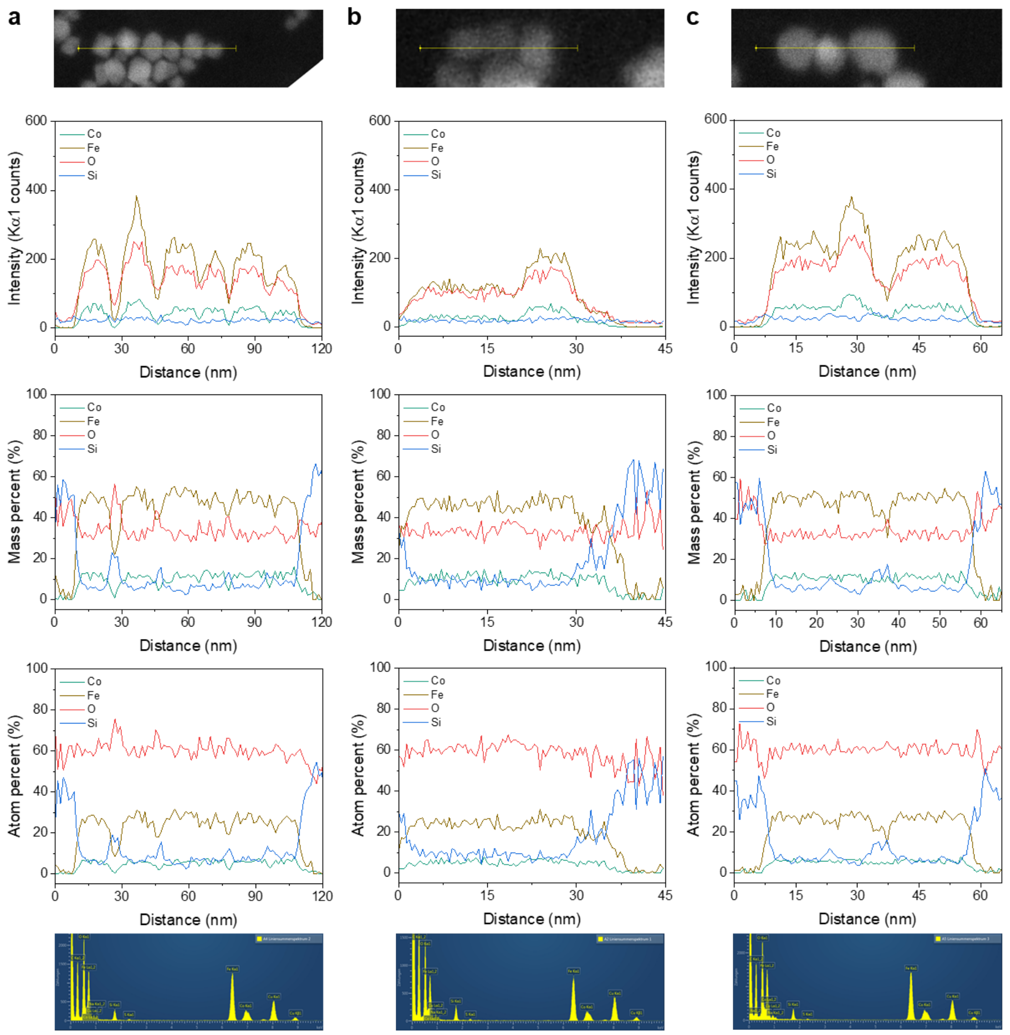

Supplementary Figure 6.7. Line scan profiles and line sum spectra of sample CF-P3-1. a-c) Three different areas show a similar line sum distribution of Co, Fe, O, and Si with (4.5 ± 0.1), (21.4 ± 1.1), (69.0 ± 0.3), and (5.1 ± 1.0) atom%, on average. However, the distribution includes only the intensities of Si, Co, Fe, and O, which were normalized to 100 mass% and 100 atom%. Therefore, the absolute values should be lower when considering all elements, as other components are not included in this normalization. The Co/Fe ratio is (0.210 ± 0.008).

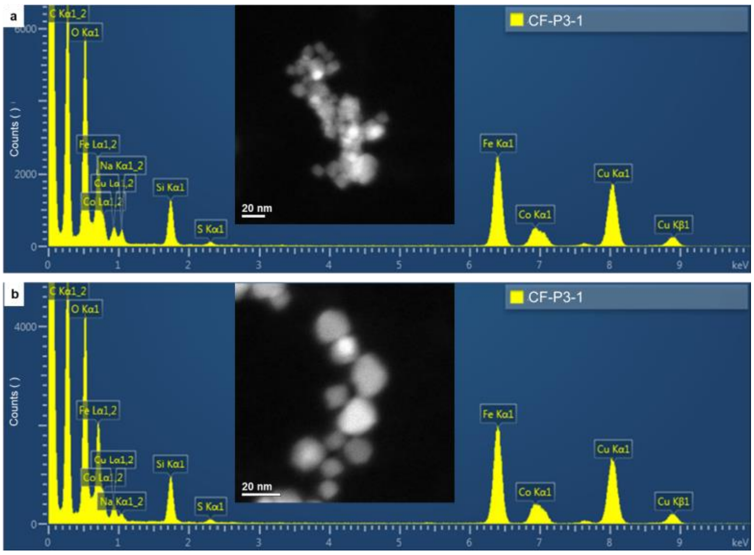

Supplementary Figure 6.8. Map sum spectra of sample CF-P3-1. Two different areas show a similar sum distribution of Co, Fe, O, and Si with (3.6 ± 0.2), (17.1 ± 0.8), (71.4 ± 1.0), and (7.9 ± 0.01) atom%, on average. The Co/Fe ratio is (0.210 ± 0.004).

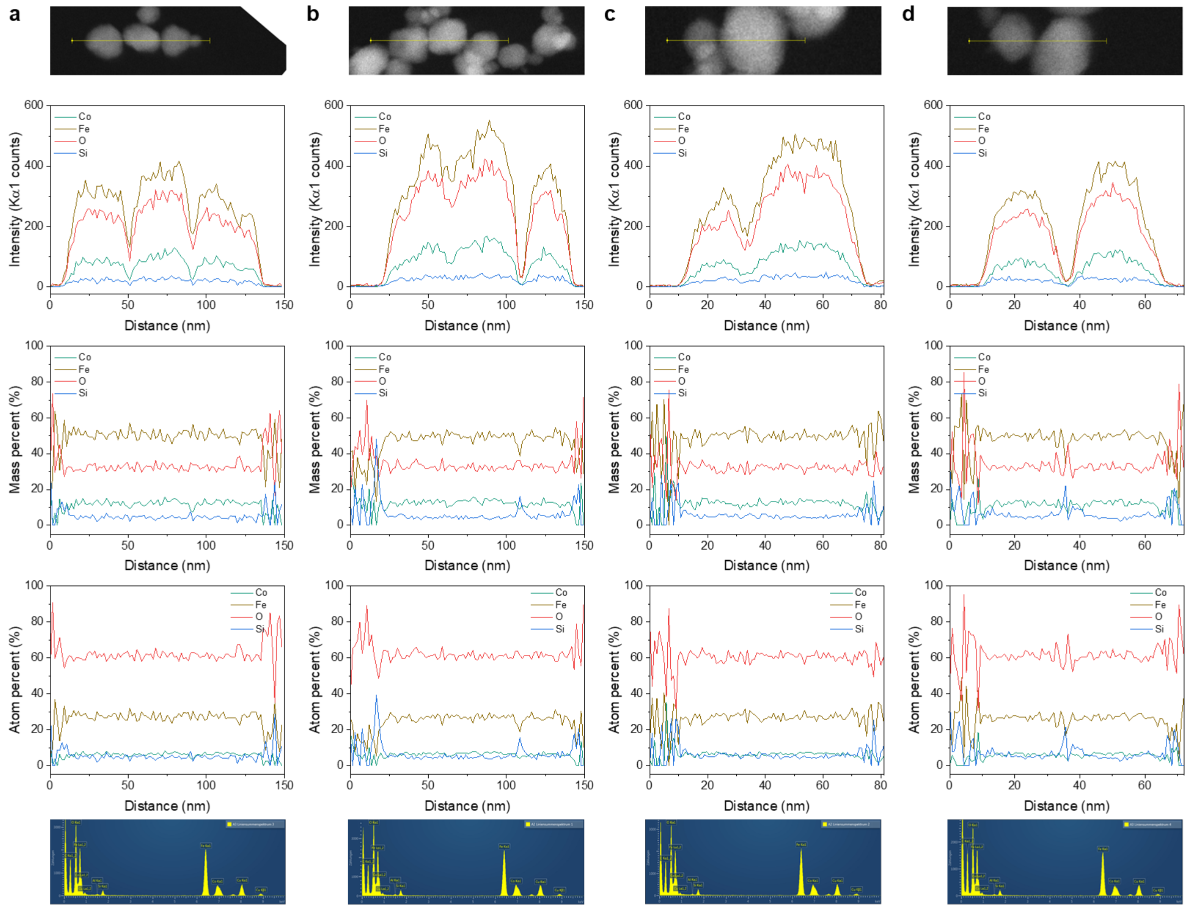

Supplementary Figure 6.9. Line scan profiles and line sum spectra of sample CF-S3-8. a-d) Four different areas show a similar line sum distribution of Co, Fe, O, and Si with (5.5 ± 0.2), (23.3 ± 0.2), (68.8 ± 0.3), and (2.4 ± 0.1) atom%, on average. The line scans indicate that thin silica shells have formed around the particles, with local silicon concentrations of 10 – 40 atom%. This is particularly evident in the areas depicted in **b** and **c**. However, the distribution includes only the intensities of Si, Co, Fe, and O, which were normalized to 100 mass% and 100 atom%. Therefore, the absolute values should be lower when considering all elements, as other components are not included in this normalization. The Co/Fe ratio is (0.236 ± 0.009).

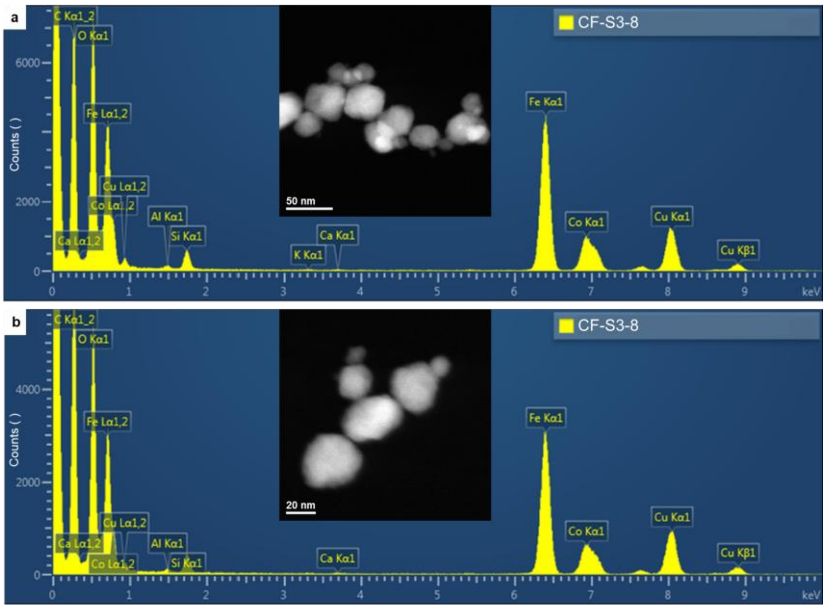

Supplementary Figure 6.10. Map sum spectra of sample CF-S3-8. Two different areas show a similar sum distribution of Co, Fe, O, and Si with (5.1 ± 0.2), (22.3 ± 0.3), (70.0 ± 0.3), and (2.7 ± 0.2) atom%, on average. The Co/Fe ratio is (0.227 ± 0.006).

S7. Size-Dependent Synthesis and Arrangement of Cobalt Ferrite Chains: Mechanism and Summarized Effects

Supplementary Note 7.1. Calculation of the Theoretical Cobalt-to-Iron Ratio

Assuming that the total amount of akaganeite (0.619 mmol) is completely converted into magnetite with the participation of Fe(II) ions (0.416 mmol), a maximum of magnetite (M) with the formula $FeO \cdot Fe_2O_3$ can be formed. The iron content can be calculated using Supplementary Equation 7.1:

$$n_{M,Aka}(Fe) = n(\text{akaganeite}) + n(Fe^{2+}) \quad 7.1$$

Considering the maximum applicable amounts of akaganeite, the iron content yields the following proportionality (Supplementary Equation 7.2):

$$n_{max}(Fe^{2+}) = \frac{n(\text{akaganeite})}{2} = 0.310 \text{ mmol} \quad 7.2$$

This results in:

$$n_{M,Aka}(Fe) = 0.929 \text{ mmol}$$

Additionally, magnetite can be generated from (the excess of) Fe(III) and Fe(II) ions at room temperature via basic precipitation. The maximum amount of magnetite by both processes - restricted by the amount of Fe(II) ions – can calculated as:

$$n_M(Fe) = n(Fe^{3+}) + n(\text{aka}) + n(Fe^{2+}) = 3\, n_{\text{weight-in}}(Fe^{2+}) \quad 7.3$$

$$n_M(Fe) = (0.832 + 0.416) \text{ mmol} = 1.248 \text{ mmol}. \quad 7.4$$

Furthermore, the iron content of a cobalt-doped (CF) phase synthesized *via* cobalt ion exchange reaction can be calculated *via* Supplementary Equation 7.5:

$$n_{CF}(Fe) = n_M(Fe) - n(Co^{2+}) \quad 7.5$$

Under the assumption of a complete exchange of the existing Co(II) ions (0.208 mmol) with Fe(II) ions to a cobalt-doped ferrite phase, the iron content is

$$n_{CF}(Fe) = (1.248 - 0.208) \text{ mmol} = 1.040 \text{ mmol}, \quad 7.6$$

which leads to a maximum cobalt-to-iron ratio κ of 0.20 (Supplementary Equation 7.7):

$$\kappa = \frac{n_{CF}(Co)}{n_{CF}(Fe)} = \frac{n(Co^{2+})}{n_{CF}(Fe)} = 0.20. \quad S7.7$$

As the exchanged Fe(II) ions can be used for additional magnetite formation steps, the cobalt-to-iron ratio would decrease and can be excluded from consideration when calculating the theoretically determined maximum cobalt content per unit volume of a cubic particle. It can also exclude that a combination of Co(II) ions and akaganeite only would form cobalt ferrite particles, as this is discussed in previous work[1,25] and would not lead to the formation of cobalt ferrite phases.

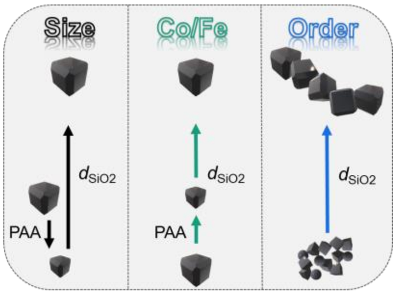

Supplementary Figure 7.1. Illustration of the summarized effects of PAA addition and silica shell on the particle size, cobalt-to-iron ratio, and chain formation.

Supplementary Section 8. Hysteresis of Self-Arranged Nanochains

Supplementary Note 8.1.

The VMS measurements of the aqueous solutions of CF-S1 and CF-S2 series are shown in Supplementary Figure 8.1a. With increasing temperature, the saturation magnetization increases slightly. However, the diameter of these samples increases in the order CF-S1-1 < CF-S2-1 < CF-S1-2 < CF-S2-2 and shows no significant trend with M_S. The samples of series 3 with a silica layer above 4 nm (Supplementary Figure 8.1b) show an increasing M_S value with the diameter. An exception is CF-S3-6, as this sample has a significantly higher cobalt content, which is associated with lower M_S values. The reference samples synthesized with bare akaganeite are depicted in Supplementary Figure 8.1c, showing no significantly lower M_S values due to the magnetite-like structure (lower cobalt content).

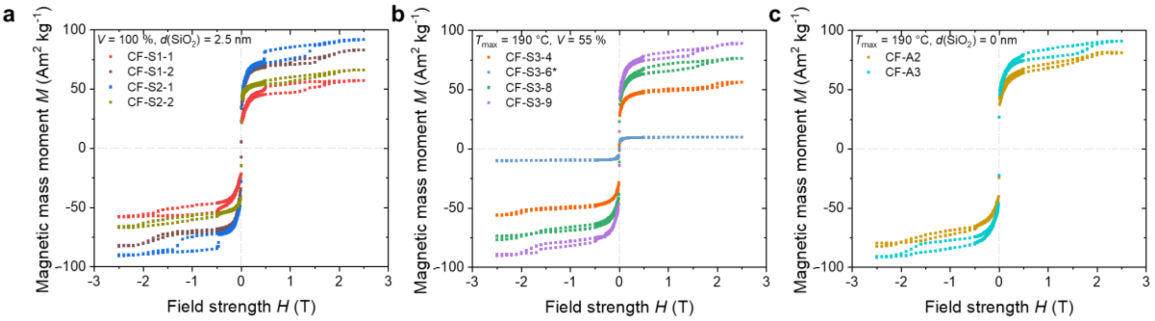

Supplementary Figure 8.1. Full-loop hysteresis curves of the CF samples dispersed in water. The samples are synthesized with the precursors **a**) aka@0.40PAA@2.5SiO_2 at different temperatures, **b**) aka@0.12PAA@SiO_2 at 190 °C with different silica thicknesses, and **c**) akaganeite at 190 °C at different filling volumes. *Sample CF-S3-6 shows a significantly higher cobalt content (x = 0.56, κ = 0.23) after the hydrothermal step, so we suggest that this sample was prepared with the wrong weight of the cobalt salt. The curves represent the average of three single measurements taken between -2.5 T and 2.5 T. The error bars are depicted as a deviation value for each measurement point.

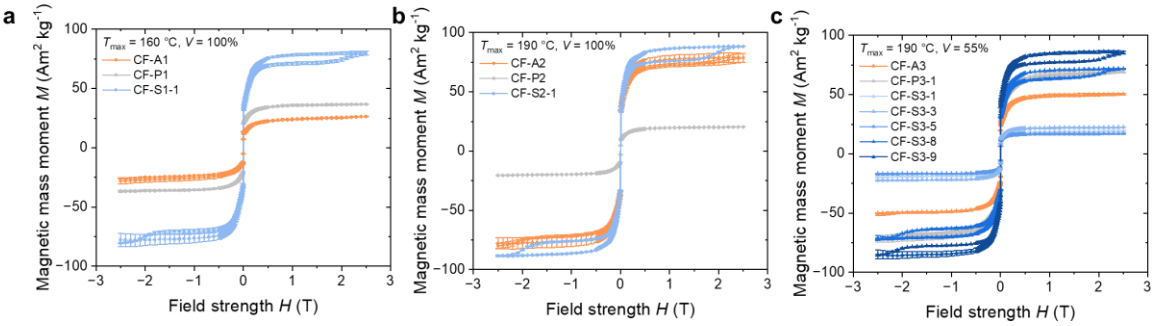

Supplementary Figure 8.2. Full-loop hysteresis curves of the CF samples dispersed in 80 wt.% glycerol/water mixture. The samples are depicted for **a**) series 1, **b**) series 2, and **c**) series 3. The curves represent the average of three single measurements taken between -2.5 T and 2.5 T. The error bars are depicted as a deviation value for each measurement point.

Supplementary Section 9. SAXS-Measurements

Supplementary Note 9.1.

The SAXS setup with two permanent magnets is depicted in Supplementary Figure 9.1 with the X-ray beam perpendicular to the magnetic field direction.

Due to the setup with a maximum gap of 220 m between the magnets, zero-field conditions are not present. At the maximum gap, the field strength measured around 3 mT. A total of 12 different field strengths were measured, corresponding to the adjusted gap size, ranging from a minimum of about 3 mT to a maximum of approximately 930 mT. Measurements were conducted using only 5% of the initial beam intensity to prevent damage to the sample from the X-ray beam. For the first two samples, CF-A3 and CF-P3-1, measurements were taken at two different exposure times of 0.005 s and 0.01 s. For the remaining samples, measurements were performed twice with an exposure time of approximately 0.002 s. A total of 5 images were captured for each measurement run. The details of these measurements are summarized in Supplementary Table 9.1. The dataset can be downloaded from https://doi.org/10.15151/ESRF-DC-2160804078.

The analysis of the SAXS data is presented exemplarily by sample CF-P3-1. In Supplementary Figure 9.2, the SAXS data for the CF-P3-1 sample are shown. For the increasing and decreasing field strengths of 3 mT, 60 mT, 200 mT, 500 mT, 750 mT, and 930 mT, a 2D pattern is displayed, respectively. On the left side, the used mask is shown for all angles, parallel, and perpendicular to the magnetic field applied.

The intensity with the unified Beaucage fits is shown in Supplementary Figures 9.3 – 9.9 for the different field strengths for (a) all field directions, (b) perpendicular to the field, and (c) parallel to the field for the samples CF-A3, CF-P3-1, CF-S3-1, CF-S3-3, CF-S3-5, CF-S3-8, and CF-S3-9, respectively. The determined data from the Beaucage fits for the samples are illustrated in these figures additionally. The cluster sizes calculated from the Beaucage fits, depending on the magnetic field direction for certain azimuthal angle ranges, are presented in Supplementary Figure 9.3k – S9.9k.

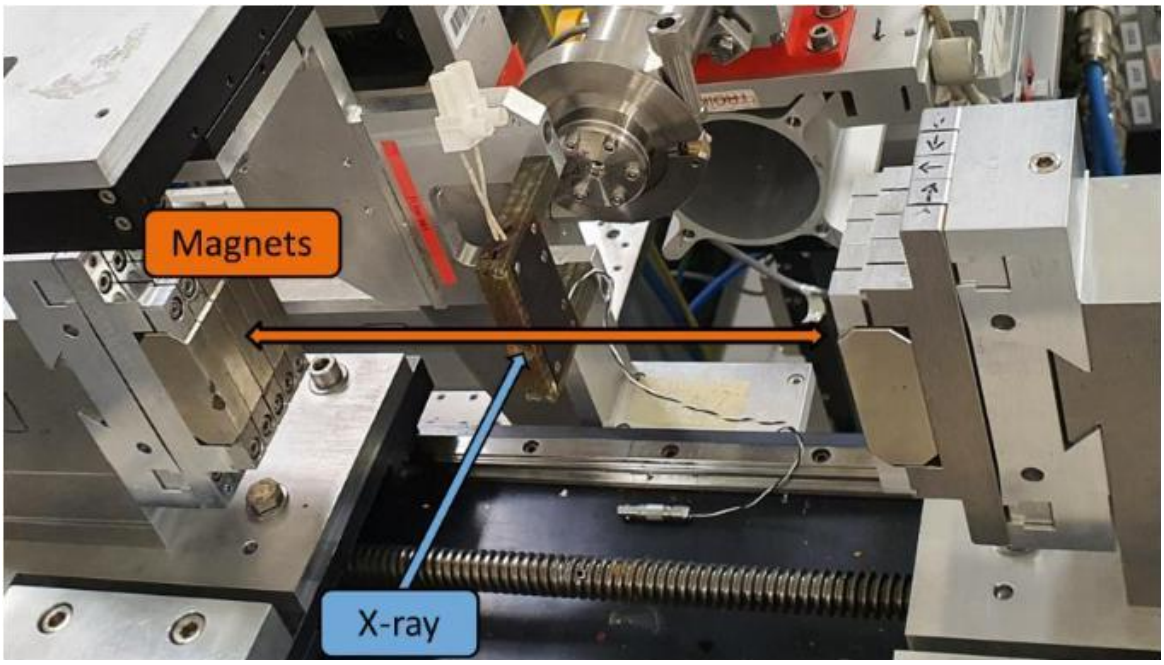

Supplementary Figure 9.1. Photo of the SAXS setup with the sample holder fixed between two magnets allowing the size of the gap to be adjusted.

Supplementary Table 9.1. Summary of experimental data for the SAXS experiment, including sample description, attenuation, experimental time, number of images, the gap between the magnets, field strength, and scan numbers.

Sample	Attenuation	Exposure time	Number of images	Gap	Calculated field strength	Scan no.	Scan no.
		(s)		(mm)	(mT)	H up	H down
**CF-A3, CF-P3-1**	0.05	0.005; 0.01	5000; 5000	220	3	1,2	45,46
				125	20	3,4	43,44
				97.6	40	5,6	41,42
				75.7	60	7,8	39,40
				63.2	80	9,10	37,38
				59.7	100	11,12	35,36
				46.2	200	13,14	33,34
				39	300	15,16	31,32
				32.5	400	17,18	29,30
				27.1	500	19,20	27,38
				19.2	750	21,22	25,26
				15.0	930	23,24	
**CF-S3-1, CF-S3-5, CF-S3-8, CF-S3-9**	0.05 ;0.1	0.002; 0.002	5000; 5000	220	3	1,2	45,46
				125	20	3,4	43,44
				97.6	40	5,6	41,42
				75.7	60	7,8	39,40
				63.2	80	9,10	37,38
				59.7	100	11,12	35,36
				46.2	200	13,14	33,34
				39	300	15,16	31,32
				32.5	400	17,18	29,30
				27.1	500	19,20	27,38
				19.2	750	21,22	25,26
				15.0	930	23,24	

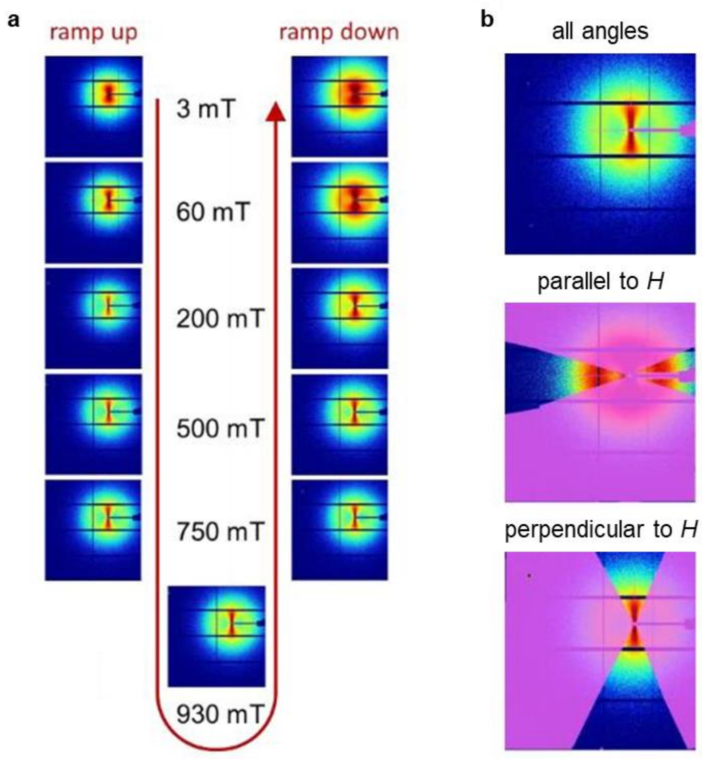

Supplementary Figure 9.2. a) 2D SAXS patterns with increasing field strength from top to bottom and decreasing field strength from bottom to top. **b**) For example, the mask is shown for all azimuthal angles, the mask parallel to the magnetic field, and the mask perpendicular to the magnetic field from top to bottom.

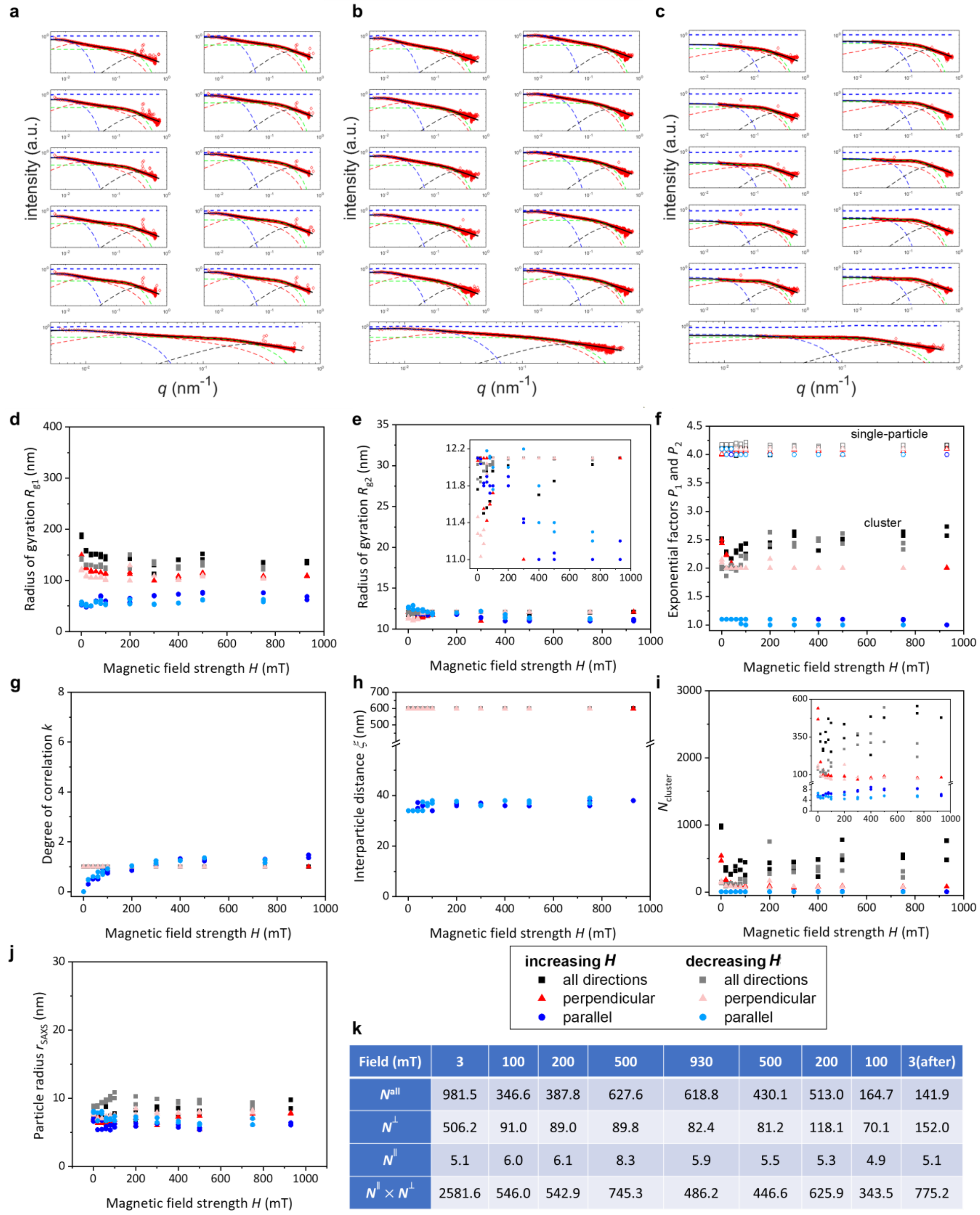

Field (mT)	3	100	200	500	930	500	200	100	3(after)
N^{all}	981.5	346.6	387.8	627.6	618.8	430.1	513.0	164.7	141.9
$N^{\perp}$	506.2	91.0	89.0	89.8	82.4	81.2	118.1	70.1	152.0
$N^{\parallel}$	5.1	6.0	6.1	8.3	5.9	5.5	5.3	4.9	5.1
$N^{\parallel} \times N^{\perp}$	2581.6	546.0	542.9	745.3	486.2	446.6	625.9	343.5	775.2

Supplementary Figure 9.3. Scattering intensity of the sample CF-A3 with the unified Beaucage fits for the increasing (left: top to bottom) and decreasing (right: bottom to top) field strengths of 3 mT, 60 mT, 200 mT, 500 mT, 750 mT, and 930 mT for **a**) all field directions, **b**) perpendicular to the field, and **c**) parallel to the field. The determined data of the Beaucage fits are depicted in dependency of **d**) the gyration radius R_{g1} of the clustered NPs, **e**) the gyration radius R_{g2} of the isolated NPs, **f**) the exponential factors P_1 and P_2, **g**) the degree of correlation k, **h**) the interparticle distance ξ, **i**) the number of NPs in a cluster, and **j**) the particle radius r_{SAXS} on the magnetic field strength. **k**) The cluster sizes $N_{\mathrm{cluster}}^{\mathrm{all}}$, $N_{\mathrm{cluster}}^{\perp}$, $N_{\mathrm{cluster}}^{\parallel}$, and the product $N_{\mathrm{cluster}}^{\perp}$ x $N_{\mathrm{cluster}}^{\parallel}$ calculated from the Beaucage fits, depending on the magnetic field strengths, are summarized.

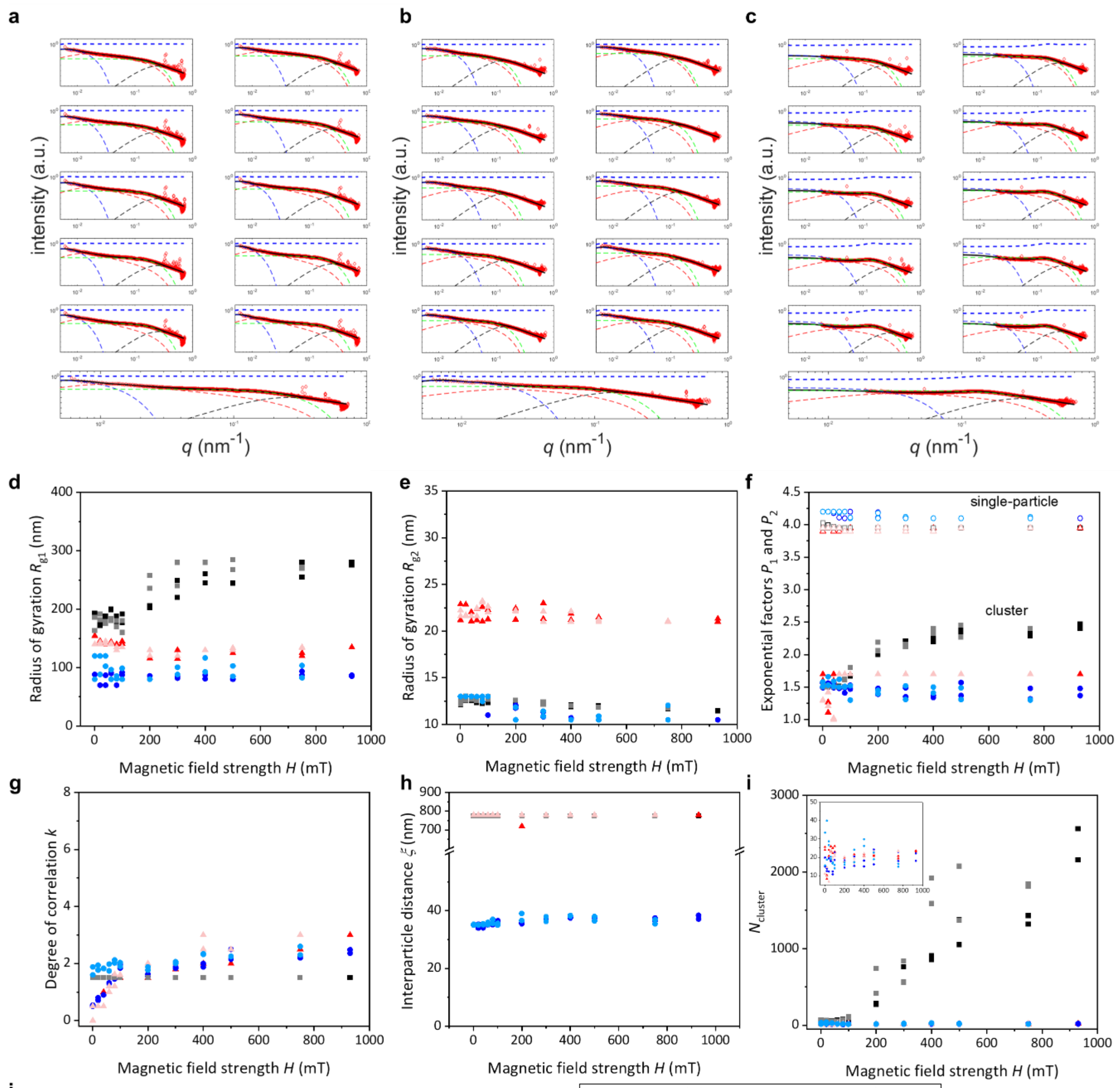

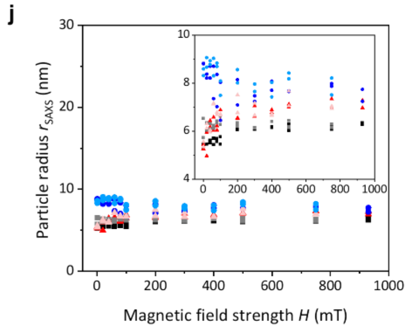

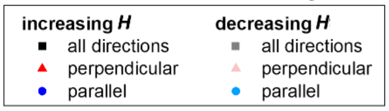

k

Field (mT)	3	100	200	500	930	500	200	100	3(after)
N^{all}	66.3	75.8	274.7	1209.0	2667.0	1709.0	578.3	104.5	56.1
$N^{\perp}$	24.9	24.6	18.9	21.1	23.3	22.5	19.0	21.3	11.0
$N^{\parallel}$	17.5	20.6	16.3	20.1	20.3	20.8	16.7	15.0	24.5
$N^{\parallel} \times N^{\perp}$	435.8	506.8	308.1	424.1	473.0	468.0	317.3	319.5	269.5

Supplementary Figure 9.4. Scattering intensity of the sample CF-P3-1 with the unified Beaucage fits for the increasing (left: top to bottom) and decreasing (right: bottom to top) field strengths of 3 mT, 60 mT, 200 mT, 500 mT, 750 mT, and 930 mT for **a**) all field directions, **b**) perpendicular to the field, and **c**) parallel to the field. The determined data of the Beaucage fits are depicted in dependency of **d**) the gyration radius R_{g1} of the clustered NPs, **e**) the gyration radius R_{g2} of the isolated NPs, **f**) the exponential factors P_1 and P_2, **g**) the degree of correlation k, **h**) the interparticle distance ξ, **i**) the number of NPs in a cluster, and **j**) the particle radius r_{SAXS} on the magnetic field strength. **k**) The cluster sizes $N_{\mathrm{cluster}}^{\mathrm{all}}$, $N_{\mathrm{cluster}}^{\perp}$, $N_{\mathrm{cluster}}^{\parallel}$, and the product $N_{\mathrm{cluster}}^{\perp}$ x $N_{\mathrm{cluster}}^{\parallel}$ calculated from the Beaucage fits, depending on the magnetic field strengths, are summarized.

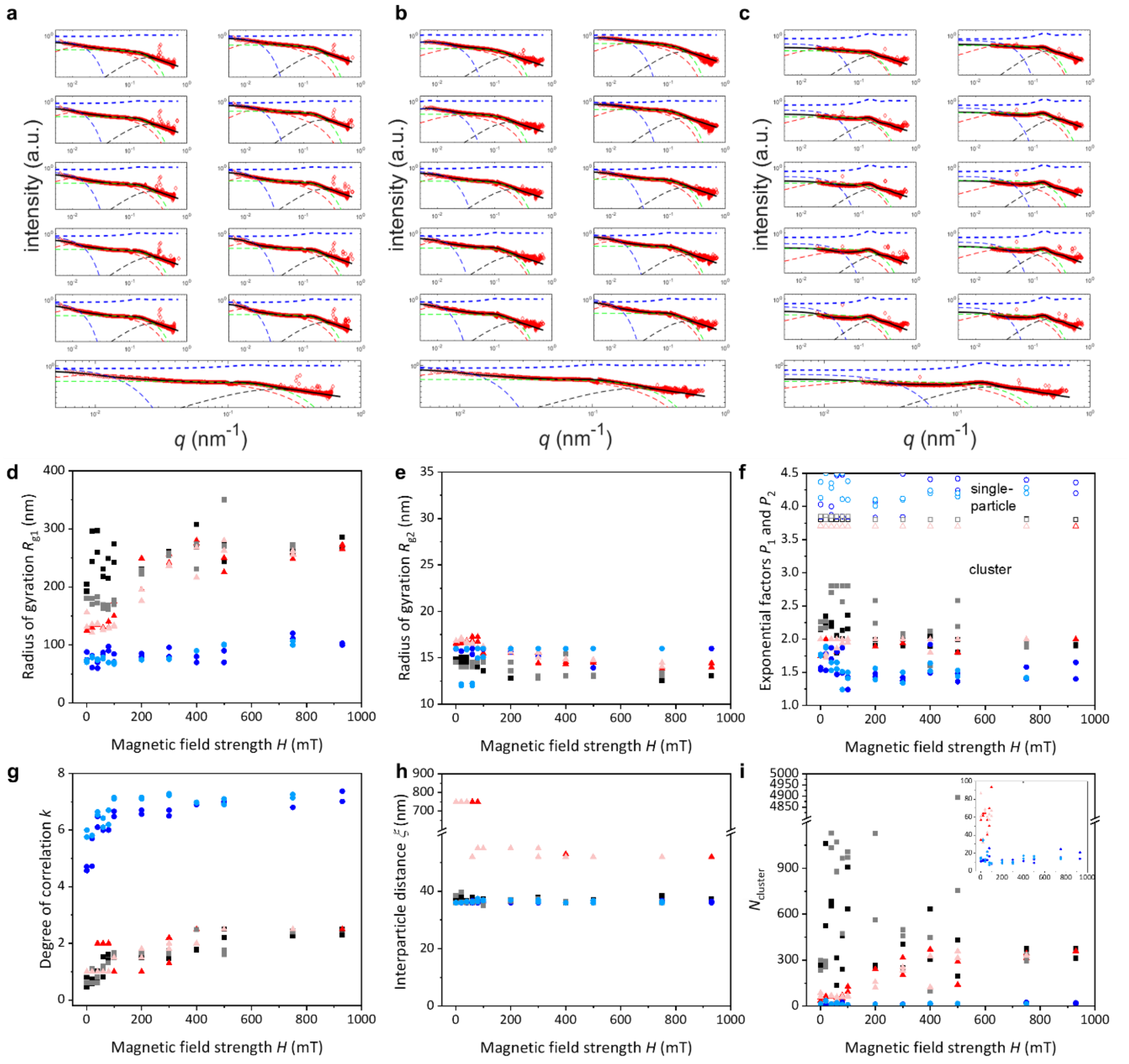

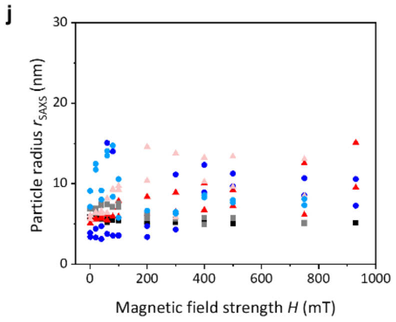

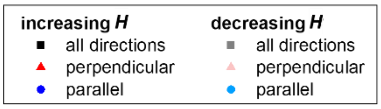

k

Field (mT)	3	100	200	500	930	500	200	100	3(after)
N^{all}	255.1	769.4	253.3	313.1	346.5	2782.0	846.4	988.4	264.8
$N^{\perp}$	45.8	110.4	181.8	215.6	357.9	336.6	138.70	64.1	73.6
$N^{\parallel}$	12.7	8.2	11.1	10.9	16.9	15.4	9.8	8.6	14.1
$N^{\parallel} \times N^{\perp}$	581.7	905	2018	2350	6049	5184	1359	551	1038

Supplementary Figure 9.5. Scattering intensity of the sample CF-S3-1 with the unified Beaucage fits for the increasing (left: top to bottom) and decreasing (right: bottom to top) field strengths of 3 mT, 60 mT, 200 mT, 500 mT, 750 mT, and 930 mT for **a**) all field directions, **b**) perpendicular to the field, and **c**) parallel to the field. The determined data of the Beaucage fits are depicted in dependency of **d**) the gyration radius R_{g1} of the clustered NPs, **e**) the gyration radius R_{g2} of the isolated NPs, **f**) the exponential factors P_1 and P_2, **g**) the degree of correlation k, **h**) the interparticle distance ξ, **i**) the number of NPs in a cluster, and **j**) the particle radius r_{SAXS} on the magnetic field strength. **k**) The cluster sizes $N^{all}_{cluster}$, $N^{\perp}_{cluster}$, $N^{\parallel}_{cluster}$, and the product $N^{\perp}_{cluster}$ x $N^{\parallel}_{cluster}$ calculated from the Beaucage fits, depending on the magnetic field strengths, are summarized.

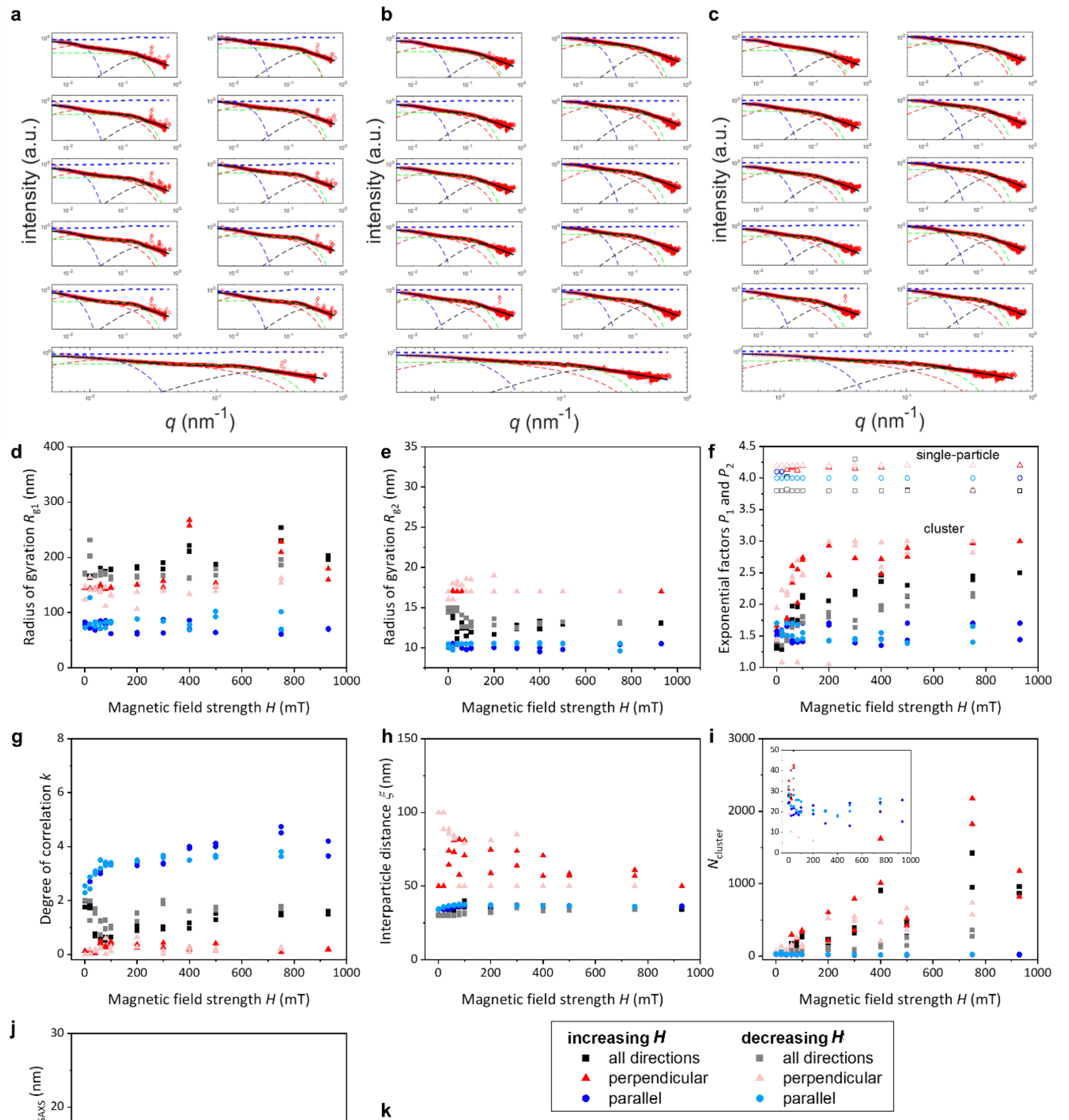

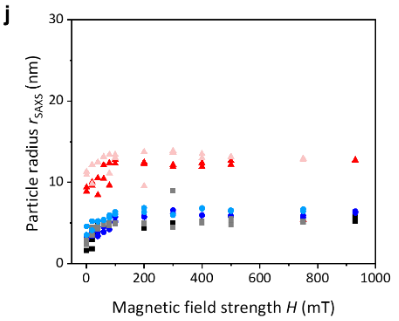

k

Field (mT)	3	100	200	500	930	500	200	100	3(after)
N^{all}	25.9	270.7	187.3	364.3	912.8	202.7	104	85.1	31.6
$N^{\perp}$	49.5	341.9	403.9	470.8	999.8	512.9	263.9	142.0	49.4
$N^{\parallel}$	24.7	21.2	21.2	18.8	20.5	21.9	20.7	22.4	28.1
$N^{\parallel} \times N^{\perp}$	1222.7	7248.3	8562.7	8851.0	20496	11233	5462.7	3180.8	1388.1

Supplementary Figure 9.6. Scattering intensity of the sample CF-S3-3 with the unified Beaucage fits for the increasing (left: top to bottom) and decreasing (right: bottom to top) field strengths of 3 mT, 60 mT, 200 mT, 500 mT, 750 mT, and 930 mT for **a**) all field directions, **b**) perpendicular to the field, and **c**) parallel to the field. The determined data of the Beaucage fits are depicted in dependency of **d**) the gyration radius R_{g1} of the clustered NPs, **e**) the gyration radius R_{g2} of the isolated NPs, **f**) the exponential factors P_1 and P_2, **g**) the degree of correlation k, **h**) the interparticle distance ξ, **i**) the number of NPs in a cluster, and **j**) the particle radius r_{SAXS} on the magnetic field strength. **k**) The cluster sizes $N^{all}_{cluster}$, $N^{\perp}_{cluster}$, $N^{\parallel}_{cluster}$, and the product $N^{\perp}_{cluster}$ x $N^{\parallel}_{cluster}$ calculated from the Beaucage fits, depending on the magnetic field strengths, are summarized.

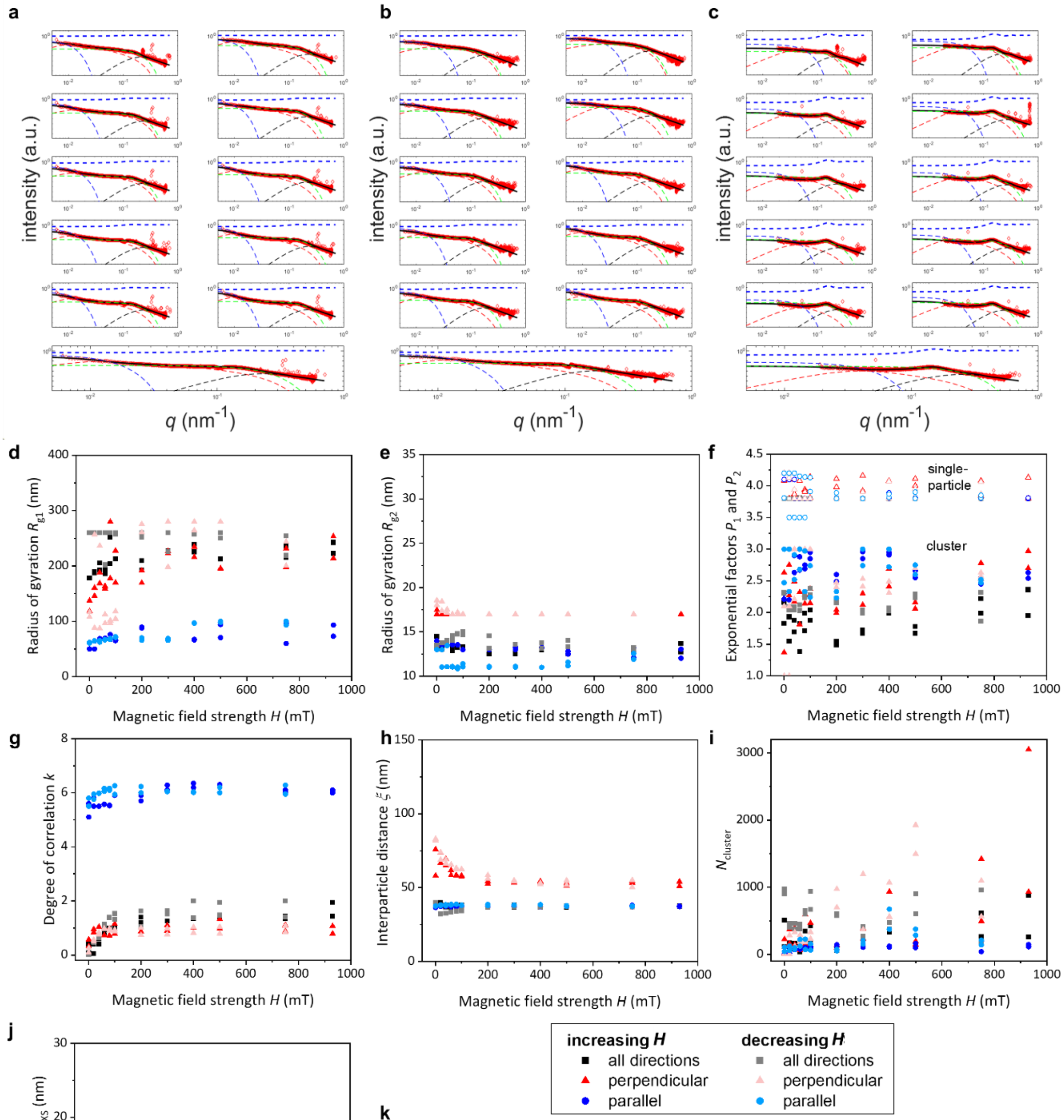

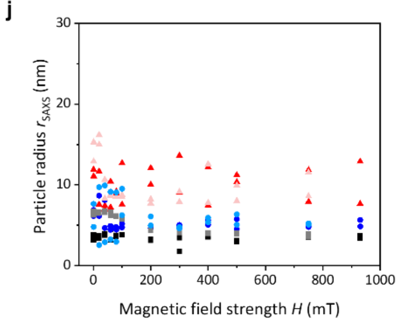

k

Field (mT)	3	100	200	500	930	500	200	100	3(after)
N^{all}	311.0	306.2	65.5	133.3	574.0	754.4	588.7	795.2	936.5
$N^{\perp}$	120.2	305.4	118.6	174.3	1994.2	1710.3	843.6	283.7	26.7
$N^{\parallel}$	18.4	110.4	132.1	131.8	131.3	327.4	63.9	111.4	76.4
$N^{\parallel} \times N^{\perp}$	2211.7	33716	15667	22973	261838	559952	53906	31604	2039.9

Supplementary Figure 9.7. Scattering intensity of the sample CF-S3-5 with the unified Beaucage fits for the increasing (left: top to bottom) and decreasing (right: bottom to top) field strengths of 3 mT, 60 mT, 200 mT, 500 mT, 750 mT, and 930 mT for **a**) all field directions, **b**) perpendicular to the field, and **c**) parallel to the field. The determined data of the Beaucage fits are depicted in dependency of **d**) the gyration radius R_{g1} of the clustered NPs, **e**) the gyration radius R_{g2} of the isolated NPs, **f**) the exponential factors P_1 and P_2, **g**) the degree of correlation k, **h**) the interparticle distance ξ, **i**) the number of NPs in a cluster, and **j**) the particle radius r_{SAXS} on the magnetic field strength. **k**) The cluster sizes $N^{all}_{cluster}$, $N^{\perp}_{cluster}$, $N^{\parallel}_{cluster}$, and the product $N^{\perp}_{cluster}$ x $N^{\parallel}_{cluster}$ calculated from the Beaucage fits, depending on the magnetic field strengths, are summarized.

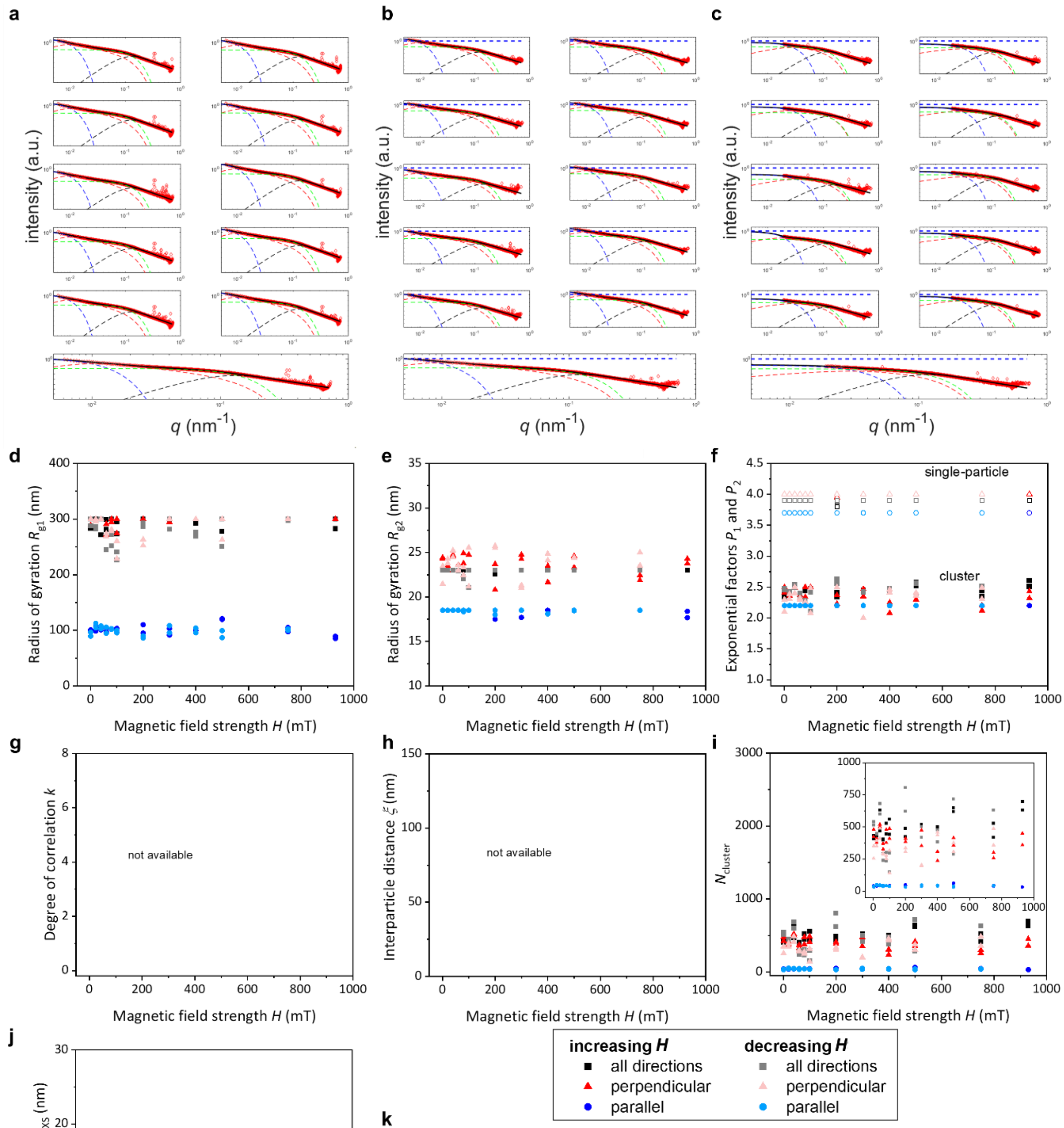

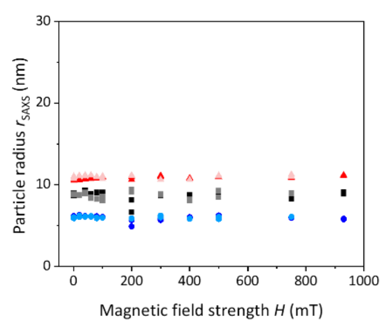

k

Field (mT)	3	100	200	500	930	500	200	100	3(after)
N^{all}	422.6	499.6	455.4	633.4	661.6	499.8	711.7	221.8	522.7
$N^{\perp}$	450.4	447.9	400.2	384.2	405.8	342.2	321.9	228.3	302.9
$N^{\parallel}$	40.8	41.3	46.0	61.7	32.1	35.3	31.9	39.0	35.0
$N^{\parallel} \times N^{\perp}$	18376	18498	18409	23705	13026	12080	10269	8904	10602

Supplementary Figure 9.8. Scattering intensity of the sample CF-S3-8 with the unified Beaucage fits for the increasing (left: top to bottom) and decreasing (right: bottom to top) field strengths of 3 mT, 60 mT, 200 mT, 500 mT, 750 mT, and 930 mT for **a**) all field directions, **b**) perpendicular to the field, and **c**) parallel to the field. The determined data of the Beaucage fits are depicted in dependency of **d**) the gyration radius R_{g1} of the clustered NPs, **e**) the gyration radius R_{g2} of the isolated NPs, **f**) the exponential factors P_1 and P_2, **g**) the degree of correlation k, **h**) the interparticle distance ξ, **i**) the number of NPs in a cluster, and **j**) the particle radius r_{SAXS} on the magnetic field strength. **k**) The cluster sizes $N^{all}_{cluster}$, $N^{\perp}_{cluster}$, $N^{\parallel}_{cluster}$, and the product $N^{\perp}_{cluster}$ x $N^{\parallel}_{cluster}$ calculated from the Beaucage fits, depending on the magnetic field strengths, are summarized. As the degree of correlation is zero for all magnetic field strengths, no magnetic-field dependency is given. This means that the interparticle distance value cannot be determined also.

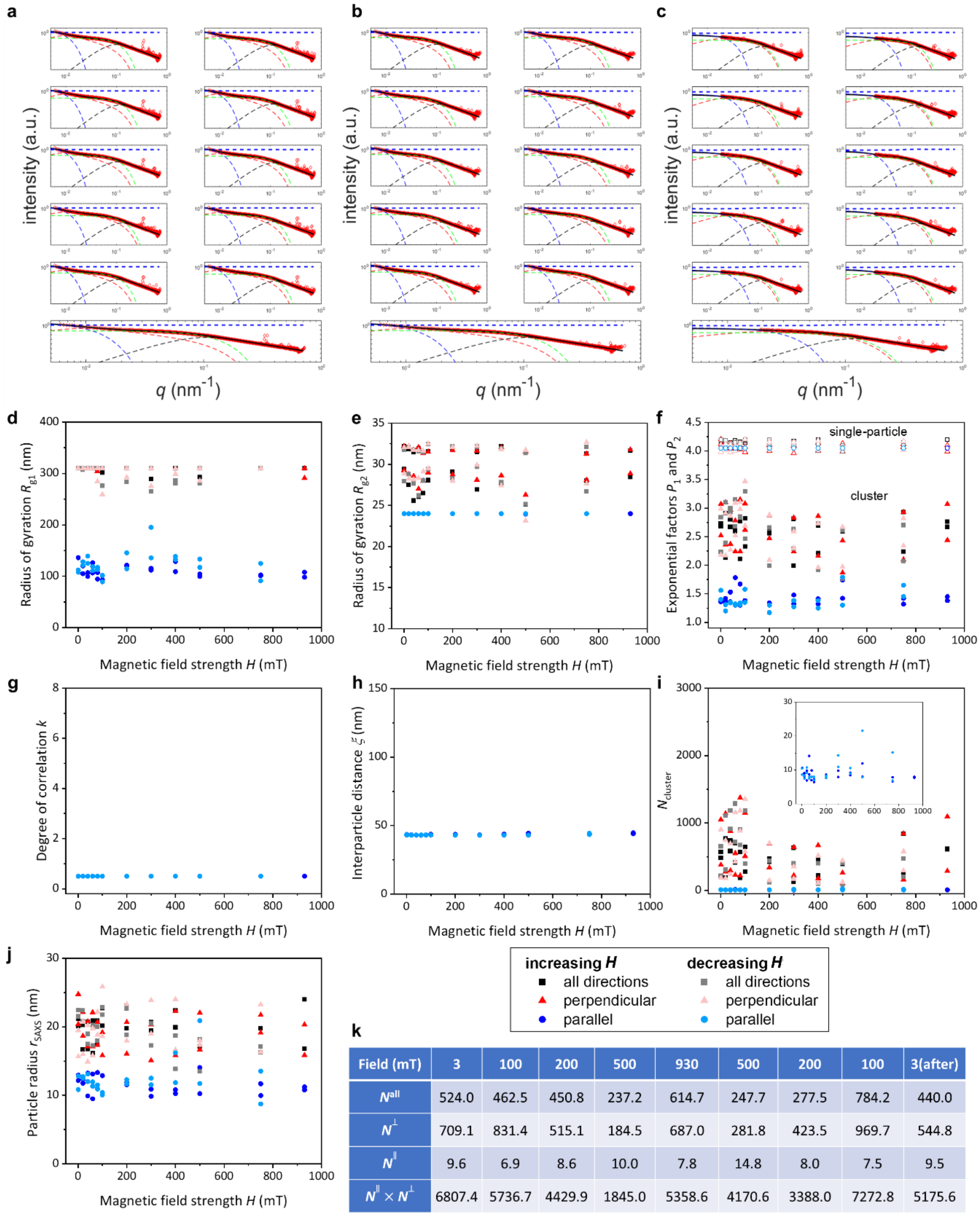

Field (mT)	3	100	200	500	930	500	200	100	3(after)
N^{all}	524.0	462.5	450.8	237.2	614.7	247.7	277.5	784.2	440.0
$N^{\perp}$	709.1	831.4	515.1	184.5	687.0	281.8	423.5	969.7	544.8
$N^{\parallel}$	9.6	6.9	8.6	10.0	7.8	14.8	8.0	7.5	9.5
$N^{\parallel} \times N^{\perp}$	6807.4	5736.7	4429.9	1845.0	5358.6	4170.6	3388.0	7272.8	5175.6

Supplementary Figure 9.9. Scattering intensity of the sample CF-S3-9 with the unified Beaucage fits for the increasing (left: top to bottom) and decreasing (right: bottom to top) field strengths of 3 mT, 60 mT, 200 mT, 500 mT, 750 mT, and 930 mT for **a**) all field directions, **b**) perpendicular to the field, and **c**) parallel to the field. The determined data of the Beaucage fits are depicted in dependency of **d**) the gyration radius R_{g1} of the clustered NPs, **e**) the gyration radius R_{g2} of the isolated NPs, **f**) the exponential factors P_1 and P_2, **g**) the degree of correlation k, **h**) the interparticle distance ξ, **i**) the number of NPs in a cluster, and **j**) the particle radius r_{SAXS} on the magnetic field strength. **k**) The cluster sizes $N_{\mathrm{cluster}}^{\mathrm{all}}$, $N_{\mathrm{cluster}}^{\perp}$, $N_{\mathrm{cluster}}^{\parallel}$, and the product $N_{\mathrm{cluster}}^{\perp}$ x $N_{\mathrm{cluster}}^{\parallel}$ calculated from the Beaucage fits, depending on the magnetic field strengths, are summarized.

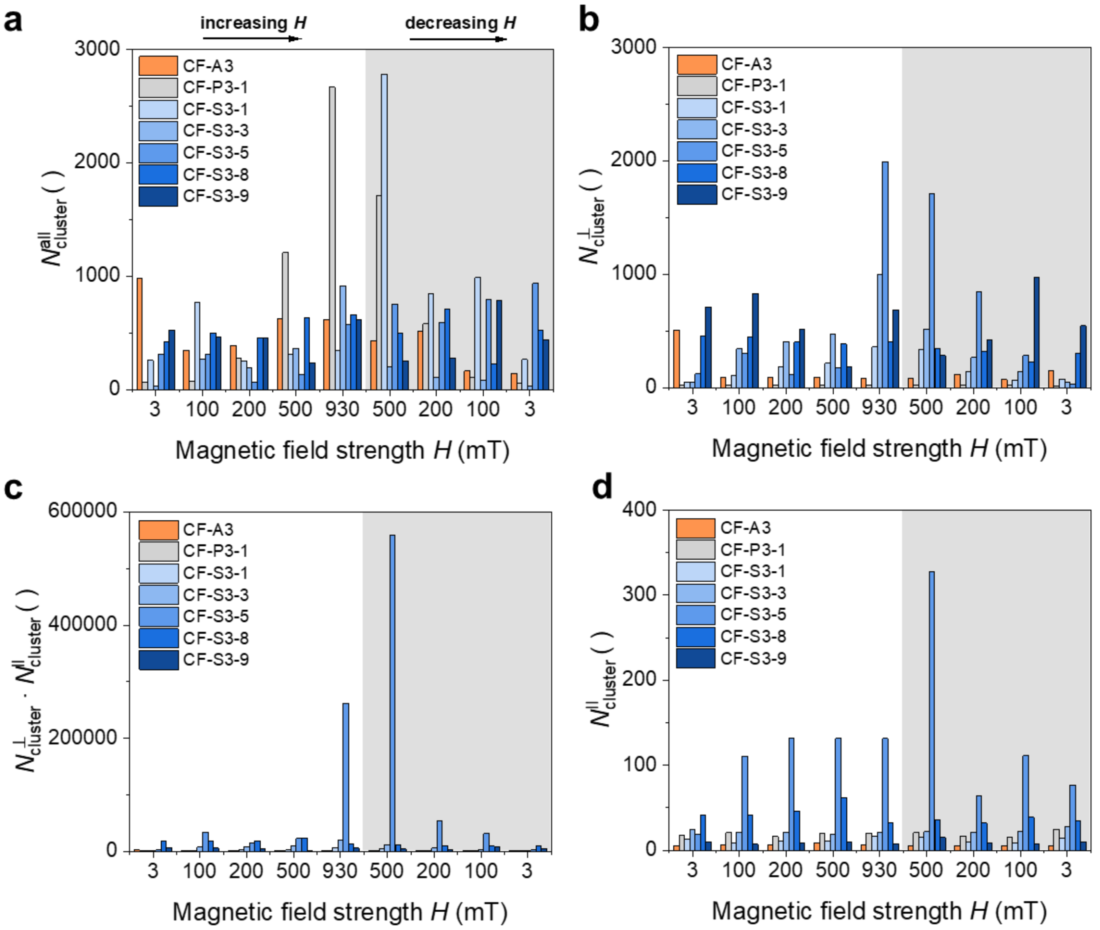

Supplementary Figure 9.10. The particle numbers of the clusters are depicted in dependency on the magnetic field with increasing and decreasing field strength for **a**) the average of all field directions ($N_{\mathrm{cluster}}^{\mathrm{all}}$), **b**) averaged angles in perpendicular field direction ($N_{\mathrm{cluster}}^{\perp}$), **d**) averaged angles parallel to the field direction ($N_{\mathrm{cluster}}^{\parallel}$), and **c**) the product of $N_{\mathrm{cluster}}^{\perp}$ and $N_{\mathrm{cluster}}^{\parallel}$. $N_{\mathrm{cluster}}^{\mathrm{all}}$ increases to values of approx. 3000 NPs with increasing field strength up to 930 mT and decreases with decreasing *H* of the field again. The values of $N_{\mathrm{cluster}}^{\parallel}$ are nearly constant for each sample except for CF-S3-5, which shows the highest ratio of morphological size to the magnetic domain size. However, $N_{\mathrm{cluster}}^{\perp}$ reach significantly higher values for the samples synthesized with a silica layer.

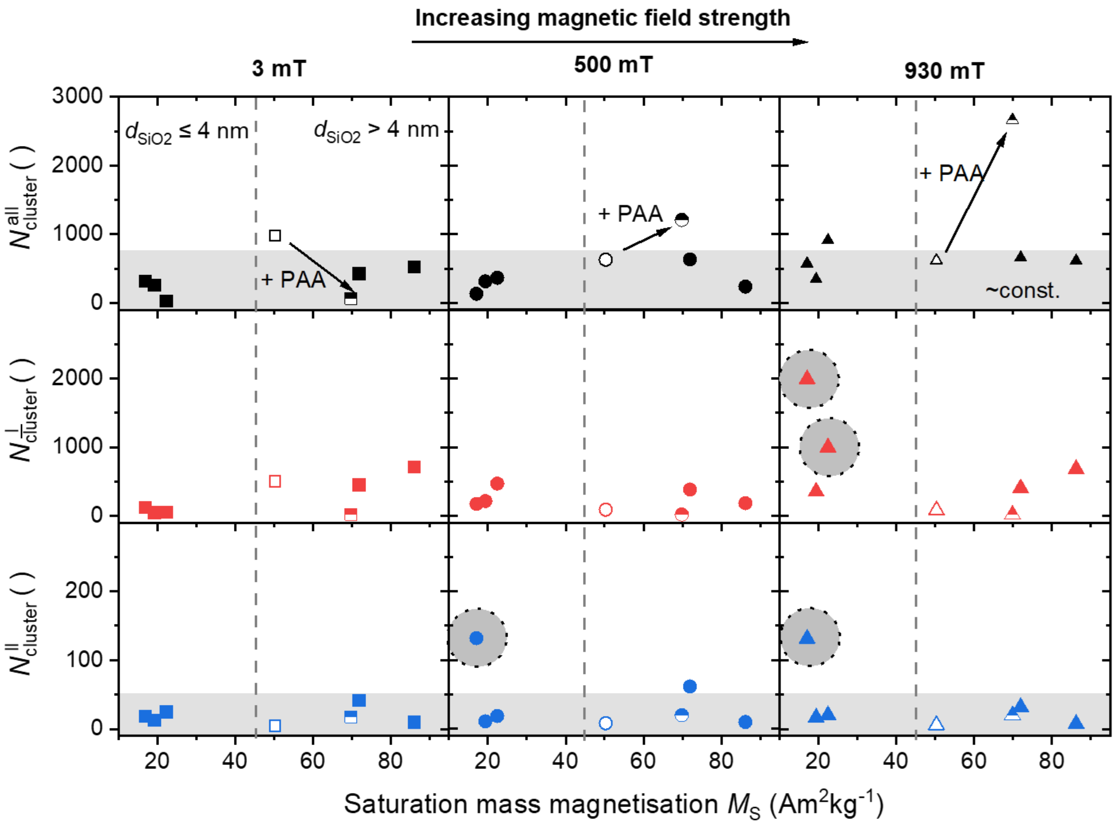

Supplementary Figure 9.11. Clustering of the particle agglomerates with increasing magnetic field strength in dependency on the mass magnetization.

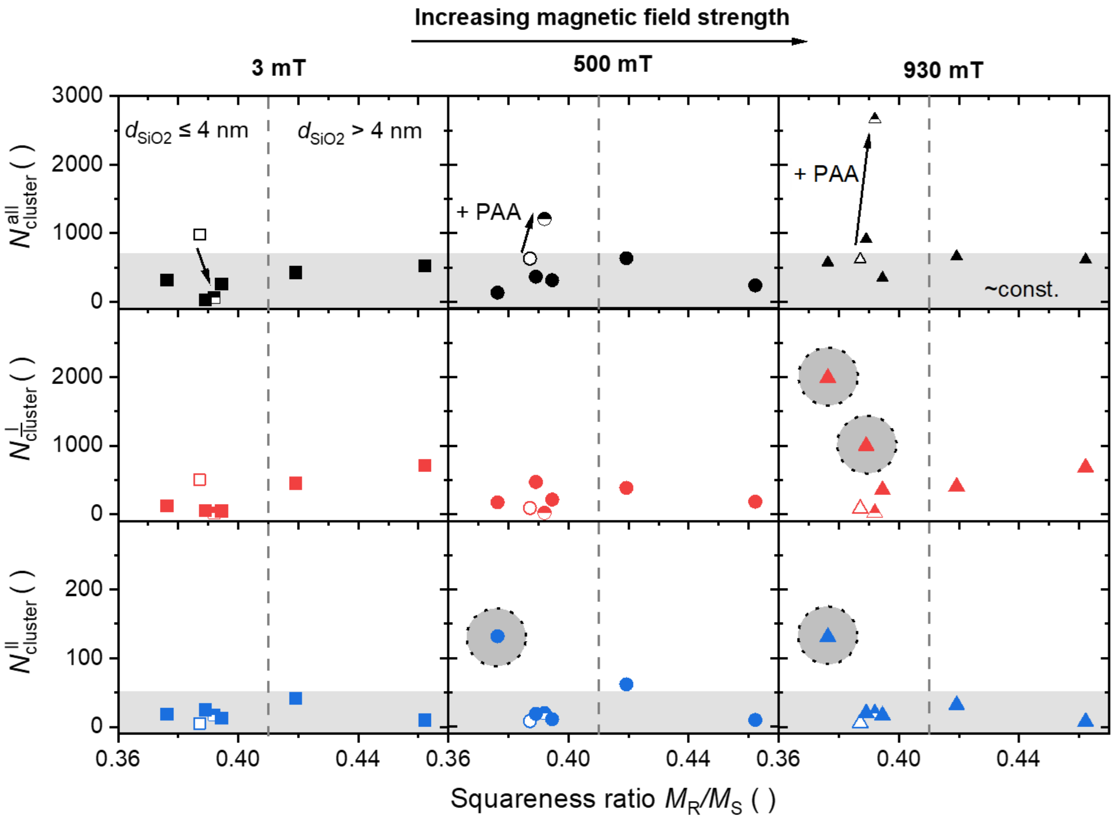

Supplementary Figure 9.12. Clustering of the particle agglomerates with increasing magnetic field strength in dependency on the squareness ratio.

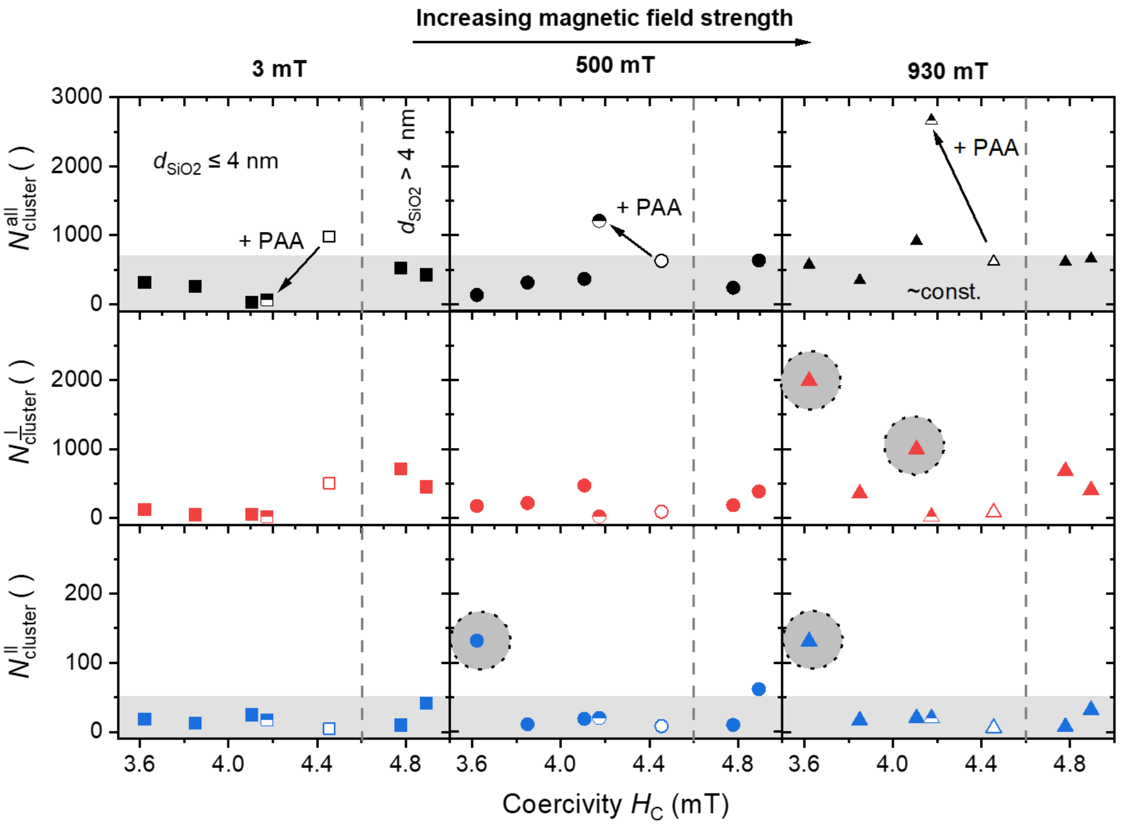

Supplementary Figure 9.13. Clustering of the particle agglomerates with increasing magnetic field strength in dependency on the coercivity.

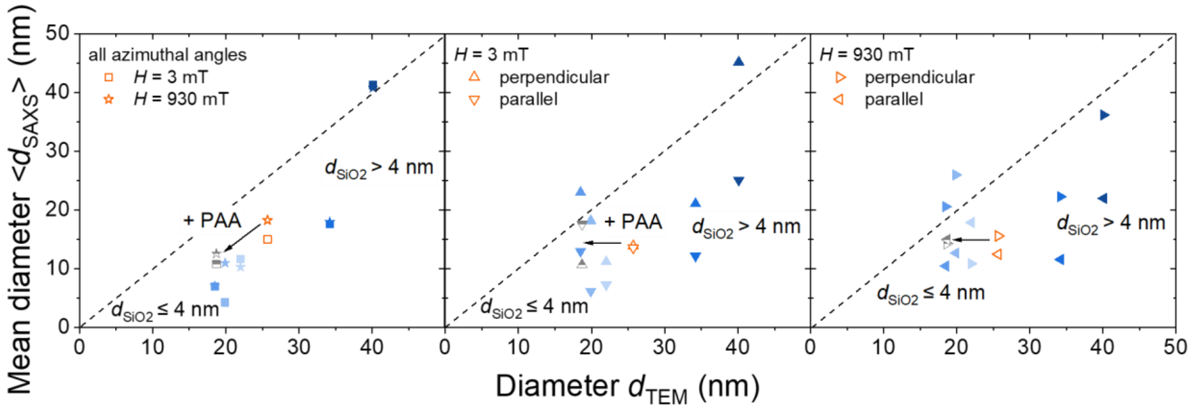

Supplementary Figure 9.14. The mean particle diameters calculated over all azimuthal angles at two field strengths and perpendicular and parallel to the beam at 3 mT and 930 mT are comparable in the trend behavior obtained *via* XRD and VSM, although d_{SAXS} is mostly slightly smaller than d_{TEM}.

Supplementary Section 10. Additional Experimental Data

In this section, the parameters of the akaganeite preparation (Supplementary Table 10.1), the PAA-modification (Supplementary Table 10.2), and the silica layer synthesis (Supplementary Table 10.3) are summarized. Furthermore, the reactor synthesis is described in Supplementary Table 10.4 with all masses of the metal salts and the used precursors.

Supplementary Table 10.1. Parameters of the akaganeite nanorod preparation.

**Sample**	**$FeCl_3 \cdot 6\ H_2O$**		**$Na_2HPO_4 \cdot 2\ H_2O$**		**Volume (DI water)**	**Dimension**	**Surface area**
	n_{FeCl3}	m_{FeCl3}	$n_{Na2HPO4}$	$V_{Na2HPO4}$	V_{DIW}	**Length x width**	$A_{O,aka}$
	**(mmol)**	**(g)**	**(mmol)**	**(mL)**	**(L)**	**(nm x nm)**	**(nm²)**
**aka1**	7.1	1.9350	0.14	3.20	0.350	(31 ± 8) nm x (7 ± 2)	750
**aka2**	10.2	2.7729	0.20	4.60	0.500	(33 ± 9) nm x (7 ± 2)	800

Supplementary Table 10.2. Synthesis parameters of the PAA-modification.

**Sample**	**Mass (PAA)**	**Volume (DI water)**	**Mass (aka)**	**Volume (stock solution) after washing steps**
	m_{PAA}	V_{DIW}	m_{aka}	$V_{stock\ solution}$
	**(mg)**	**(mL)**	**(mg)**	**(mL)**
**aka2@0.04PAA-1**	6.6	174	110	1.75
**aka2@0.04PAA-2**	6.6	174	110	1.75
**aka2@0.04PAA-3**	6.6	174	110	for analysis only
**aka1@0.12PAA-1**	22.8	174	110	1.75
**aka2@0.12PAA-2**	23.8	174	110	1.75
**aka2@0.12PAA-3**	22.4	174	110	*for analysis only*
**aka2@0.12PAA-4**	22.3	174	110	1.75
**aka2@0.12PAA-5**	125.5	870	550	8.75
**aka2@0.12PAA-6**	125.7	870	550	8.75
**aka1@0.40PAA-1**	367.7	870	550	8.75

Supplementary Table 10.3. Synthesis parameters of the silica modification.

Sample	PAA Sample	Volume (stock solution)	Volume (ethanol)	Volume ($NH_{3(aq)}$)	Volume of TEOS per iteration	Number of iterations	Amount of TEOS per surface area of all particles	Amount of TEOS per surface area and concentration	Silica layer thickness	
		$V_{stock\ solution}$	V_{EtOH}	V_{NH3}	V_{TEOS}/N	N	$n_{TEOS}/A_{O,total}$	$n_{TEOS}/(A_{O,total} \cdot V_{Solution})$	d_{SiO2}	σ_{SiO2}
		(mL)	(mL)	(µL)	(µL)	( )	(10^{-28} mmol nm^{-2})	(10^{-28} mmol nm^{-2} L^{-1})	(nm)	(nm)
**S4-1**	aka2@0.04PAA-1	0.875	20	125	12.4	1	1.9	91	2.5	1.1
**S4-2**	aka2@0.04PAA-1	0.875	20	125	24.8	2	9.1	435	3.6	0.9
**S4-3**	aka2@0.04PAA-2	0.875	40	250	10.1	2	3.1	74	3.1	0.9
**S4-4**	aka2@0.04PAA-2	0.875	20	250	12.1	2	3.7	177	5.0	0.9
**S3-1**	aka2@0.12PAA-6	1.750	40	250	5.7	1	0.4	10	2.2	1.5
**S3-2***	aka2@0.12PAA-2	1.750	40	250	5.7	1	0.4	10	2.6	0.9
**S3-3**	aka2@0.12PAA-5	0.875	20	125	11.4	1	1.7	83	3.4	1.7
**S3-4**	aka2@0.12PAA-6	0.875	20	125	11.4	1	1.7	83	3.7	1.7
**S3-5**	aka2@0.12PAA-6	0.875	20	125	22.8	2	7.0	333	4.0	1.6
**S3-6**	aka2@0.12PAA-4	1.750	40	250	22.8	1	1.7	42	4.6	1.8
**S3-7***	aka2@0.12PAA-5	0.875	20	125	22.8	2	7.0	333	5.3	2.1
**S3-8**	aka1@0.12PAA-1	0.875	20	125	11.4	1	1.9	89	5.6	2.4
**S3-9**	aka1@0.12PAA-1	0.875	20	125	22.8	2	7.5	358	6.8	1.9
**S1-1, S1-2, S2-1, S2-2**	aka1@0.40PAA-1	7.000	160	1000	236.8	1	4.8	29	2.5	3.2

*Samples S3-2 and S3-7 were not used for the hydrothermal reactions as the stability decreased after two weeks. All other silica-modified samples with 0.12 mg/mL PAA were used immediately after synthesis.

Supplementary Table 10.4. Reactor parameters of the cobalt ferrite synthesis. CF samples were synthesized with different precursors (bare akaganeite (A), PAA-modified akaganeite (P), or silica-functionalized akaganeite (S)), filling volumes, and temperatures.

**Sample**	**Precursor**			**Metal salts**						**Volume (DI Water)**	**Maximum temperature**	**Filling ratio**
	**Sample**	n	V	n_{CoCl2}	n_{FeCl2}	n_{FeCl3}	m_{CoCl2}	m_{FeCl2}	m_{FeCl3}	V_{DIW}	T_{max}	V
		**(mmol)**	**(mL)**	**(mmol)**	**(mmol)**	**(mmol)**	**(mg)**	**(mg)**	**(mg)**	**(mL)**	**(°C)**	**(%)**
**CF-A1**	aka2	1.125	10.0	0.377	0.759	1.116	89.7	150.9	301.6	2.5	160	100
**CF-P1**	aka2@0.12PAA-6	1.125	5.8	0.378	0,757	1.117	89.9	150.6	302.0	6.7	160	100
**CF-S1-1**	S1-1	1.125	6.8	0.377	0.758	1.115	89.8	150.7	301.5	5.7	160	100
**CF-S1-2**	S1-2	1.125	6.8	0.376	0.759	1.121	89.5	150.8	302.9	5.7	160	100
**CF-A2**	aka1	1.125	9.1	0.379	0.759	1.115	90.2	150.8	301.5	3.4	190	100
**CF-P2**	aka2@0.12PAA-6	1.125	4.5	0.381	0.759	1.116	90.7	150.8	301.6	8.0	190	100
**CF-S2-1**	S2-1	1.125	6.8	0.415	0.756	1.115	98.7	150.3	301.4	5.7	190	100
**CF-S2-2**	S2-1	1.125	6.8	0.377	0.764	1.114	89.6	151.9	301.0	5.7	190	100
**CF-A3**	aka1	0.619	5.0	0.208	0.418	0.613	49.5	83.1	165.8	1.9	190	55
**CF-P3-1**	aka2@0.12PAA-5	0.619	3.2	0.208	0.417	0.612	49.5	82.9	165.5	3.7	190	55
**CF-P3-2**	aka2@0.12PAA-6	0.619	3.2	0.210	0.419	0.616	49.9	83.4	166.5	3.7	190	55
**CF-S3-1**	S3-1	0.619	1.0	0.212	0.418	0.615	50.5	83.2	166.2	5.9	190	55
**CF-S3-3**	S3-3	0.619	2.0	0.211	0.419	0.611	50.2	83.3	165.1	4.9	190	55
**CF-S3-4**	S3-4	0.619	4.6	0.207	0.413	0.615	49.3	82.2	166.1	2.3	190	55
**CF-S3-5**	S3-5	0.619	2.0	0.208	0.414	0.615	49.4	82.3	166.3	4.9	190	55
**CF-S3-6***	S3-7*	0.619	5.0	0.208	0.415	0.613	49.6	82.5	165.8	1.9	190	55
**CF-S3-8**	S3-8	0.619	0.9	0.207	0.416	0.615	49.2	82.7	166.1	6.0	190	55
**CF-S3-9**	S3-9	0.619	0.9	0.208	0.417	0.611	49.6	82.9	165.2	6.0	190	55

*Sample CF-S3-6 shows a significantly higher cobalt content (x = 0.56, κ = 0.23) after the hydrothermal step, so we suggest that this sample was prepared with the wrong weight-in of the cobalt salt.

Supplementary Section 11. References

1. Weißpflog, M., Nguyen, N., Sobania, N. & Hankiewicz, B. Non-Stoichiometric Cobalt Ferrite Nanoparticles by Green Hydrothermal Synthesis and their Potential for Hyperthermia Applications. *J. Phys. Chem. C* **128**; 10.1021/acs.jpcc.4c03589 (2024).
2. Hinrichs, S. *et al.* Goethite Nanorods: Synthesis and Investigation of the Size Effect on Their Orientation within a Magnetic Field by SAXS. *Nanomaterials (Basel, Switzerland)* **10**; 10.3390/nano10122526 (2020).
3. Ozaki, M., Kratohvil, S. & Matijević, E. Formation of monodispersed spindle-type hematite particles. *J. Colloid Interface Sci.* **102**; 10.1016/0021-9797(84)90208-X (1984).
4. Sugimoto, T., Wang, Y., Itoh, H. & Muramatsu, A. Systematic control of size, shape and internal structure of monodisperse α-Fe_2O_3 particles. *Colloids Surf. A Physicochem. Eng. Asp.* **134**; 10.1016/S0927-7757(97)00103-9 (1998).
5. Frandsen, C. *et al.* Aggregation-induced growth and transformation of β-FeOOH nanorods to micron-sized α-Fe_2O_3 spindles. *CrystEngComm* **16**; 10.1039/c3ce40983j (2014).
6. Allender, C. J. *et al.* The Role of Growth Directors in Controlling the Morphology of Hematite Nanorods. *Nanoscale Res. Lett.* **15**; 10.1186/s11671-020-03387-w (2020).
7. Milosevic, I. *et al.* Facile Microwave Process in Water for the Fabrication of Magnetic Nanorods. *J. Phys. Chem. C* **115**; 10.1021/jp205334v (2011).
8. Hijnen, N. & Clegg, P. S. Simple Synthesis of Versatile Akaganéite-Silica Core–Shell Rods. *Chem. Mater.* **24**; 10.1021/cm301772p (2012).
9. Bailey, J. K, Brinker, C. J. & Mecartney, M. L. J. Growth Mechanisms of Iron Oxide Particles of Differing Morphologies from the Forced Hydrolysis of Ferric Chloride Solutions. *J. Colloid Interface Sci.* **157**; 10.1006/jcis.1993.1150 (1993).
10. Purbia, R. & Paria, S. Green Synthesis of Single-Crystalline Akaganeite Nanorods for Peroxidase Mimic Colorimetric Sensing of Ultralow-Level Vitamin B1 and Sulfide Ions. *ACS Appl. Nano Mater.* **1**; 10.1021/acsanm.7b00390 (2018).
11. Zhao, J., Lin, W., Chang, Q., Li, W. & Lai, Y. Adsorptive characteristics of akaganeite and its environmental applications: a review. *Environ. Technol. Rev.* **1**; 10.1080/09593330.2012.701239 (2012).
12. Zhang, L., Gonçalves, A. A. S. & Jaroniec, M. Identification of preferentially exposed crystal facets by X-ray diffraction. *RSC Adv.* **10**; 10.1039/d0ra00769b (2020).
13. Almeida, T., Fay, M., Zhu, Y. & Brown, P. D. Effect of phosphate on the morphology of hydrothermally synthesised β-FeOOH and α-Fe_2O_3 nanoparticles. *J. Phys. Conf. Ser.* **241**; 10.1088/1742-6596/241/1/012045 (2010).
14. Fu, X. *et al.* Thermal stability of akaganeite and its desiccation process under conditions relevant to Mars. *Icarus* **336**; 10.1016/j.icarus.2019.113435 (2020).
15. Nemati, Z. *et al.* Improving the Heating Efficiency of Iron Oxide Nanoparticles by Tuning Their Shape and Size. *J. Phys. Chem. C* **122**; 10.1021/acs.jpcc.7b10528 (2018).

16. Laurent, S. *et al.* Magnetic iron oxide nanoparticles: synthesis, stabilization, vectorization, physicochemical characterizations, and biological applications. *Chem. Rev.* **108**; 10.1021/cr068445e (2008).

17. Spitzmüller, L. *et al.* Dissolution control and stability improvement of silica nanoparticles in aqueous media. *J. Nanopart. Res.* **25**; 10.1007/s11051-023-05688-4 (2023).

18. Szekeres, M., Tóth, J. & Dékány, I. Specific Surface Area of Stoeber Silica Determined by Various Experimental Methods. *Langmuir* **18**; 10.1021/la011370j (2002).

19. Vrij, A. *et al.* Light scattering of colloidal dispersions in non-polar solvents at finite concentrations. Silic spheres as model particles for hard-sphere interactions. *Faraday Discuss. Chem. Soc.* **76**; 10.1039/DC9837600019 (1983).

20. van Blaaderen, A., van Geest, J. & Vrij, A. Monodisperse colloidal silica spheres from tetraalkoxysilanes: Particle formation and growth mechanism. *J. Colloid Interface Sci.* **154**; 10.1016/0021-9797(92)90163-G (1992).

21. Labrosse, A. & Burneau, A. Characterization of porosity of ammonia-catalysed alkoxysilane silica. *J. Non-Cryst. Solids* **221**; 10.1016/S0022-3093(97)00414-6 (1997).

22. Li, S., Wan, Q., Qin, Z., Fu, Y. & Gu, Y. Understanding Stöber silica's pore characteristics measured by gas adsorption. *Langmuir* **31**; 10.1021/la5042103 (2015).

23. Bazuła, P. A. *et al.* Highly microporous monodisperse silica spheres synthesized by the Stöber process. *Microporous Mesoporous Mater.* **200**; 10.1016/j.micromeso.2014.07.051 (2014).

24. Fullriede, H. Silica-Nanopartikel mit speziellen Eigenschaften für die Herstellung dentaler Kompositmaterialien. Dissertation. Gottfried Wilhelm Leibniz University, Hannover, Germany (2015).

25. Weißpflog, M., Eberbeck, D. & Hankiewicz, B. Shape-directed modification of truncated octahedral to coffin-like cobalt-doped ferrite particles by changing the hydrothermal reaction conditions. *RSC Adv.* **15**; 10.1039/D5RA02233A (2025).
